# Dynamic Virtual Simulation with Real-Time Haptic Feedback for Robotic Internal Mammary Artery Harvesting

**DOI:** 10.3390/bioengineering12030285

**Published:** 2025-03-13

**Authors:** Shuo Wang, Tong Ren, Nan Cheng, Rong Wang, Li Zhang

**Affiliations:** 1 Department of Engineering Physics, Key Laboratory of Particle and Radiation Imaging, Ministry of Education, Tsinghua University, Beijing 100084, China; shuo-wan19@mails.tsinghua.edu.cn; 2 Department of Adult Cardiac Surgery, Senior Department of Cardiology, The Six Medical Center of PLA General Hospital, Fucheng Road, Haidian District, Beijing 100048, China; rentong8713@163.com (T.R.); cn86919@163.com (N.C.); 3 Chinese PLA Medical School, Fuxing Road, Haidian District, Beijing 100089, China

**Keywords:** robotic cardiac surgery, coronary artery bypass grafting, internal mammary artery, surgical training, haptic feedback

## Abstract

Coronary heart disease, a leading global cause of mortality, has witnessed significant advancement through robotic coronary artery bypass grafting (CABG), with the internal mammary artery (IMA) emerging as the preferred “golden conduit” for its exceptional long-term patency. Despite these advances, robotic-assisted IMA harvesting remains challenging due to the absence of force feedback, complex surgical maneuvers, and proximity to the beating heart. This study introduces a novel virtual simulation platform for robotic IMA harvesting that integrates dynamic anatomical modeling and real-time haptic feedback. By incorporating a dynamic cardiac model into the surgical scene, our system precisely simulates the impact of cardiac pulsation on thoracic cavity operations. The platform features high-fidelity representations of thoracic anatomy and soft tissue deformation, underpinned by a comprehensive biomechanical framework encompassing fascia, adipose tissue, and vascular structures. Our key innovations include a topology-preserving cutting algorithm, a bidirectional tissue coupling mechanism, and dual-channel haptic feedback for electrocautery simulation. Quantitative assessment using our newly proposed Spatial Asymmetry Index (SAI) demonstrated significant behavioral adaptations to cardiac motion, with dynamic scenarios yielding superior SAI values compared to static conditions. These results validate the platform’s potential as an anatomically accurate, interactive, and computationally efficient solution for enhancing surgical skill acquisition in complex cardiac procedures.

## 1. Introduction

According to the latest statistics from the World Health Organization (WHO), coronary heart disease has consistently remained the leading cause of death globally over the past decade, accounting for 16.7% of total mortality [1]. With continuous advancements in medical technology, robotic cardiac surgery, representing the pinnacle of minimally invasive surgical techniques, has gained widespread adoption worldwide due to its proven efficacy, minimal invasiveness, and accelerated patient recovery [2]. Robotic CABG constitutes a crucial component of robotic cardiac surgical procedures. In these operations, the IMA has earned the moniker “golden conduit” due to its exceptional clinical outcomes. Research data demonstrates that the IMA, when used as a bypass graft, exhibits superior long-term patency rates, with 10-year patency rates exceeding 90% [3]. This remarkable advantage has established the IMA as the preferred conduit for coronary bypass procedures. Robotic-assisted IMA harvesting has achieved widespread recognition and implementation globally, attributed to several key technological advantages: high-definition 3D imaging systems providing superior surgical visualization, multi-degree-of-freedom robotic instruments offering enhanced dexterity and precise manipulation, and ergonomic control interfaces reducing surgeon fatigue [4]. These features collectively contribute to improved surgical outcomes and operator comfort during these complex procedures.

Robotic cardiac surgery, characterized by its master–slave operation mode, presents unique challenges due to the absence of force feedback and the requirement for complex, precise surgical maneuvers. Even surgeons with extensive experience in open and thoracoscopic procedures must overcome a steep initial learning curve. In the context of robotic-assisted minimally invasive CABG, which represents the most frequently performed robotic cardiac procedure, achieving expertise requires accumulating experience from approximately 250 cases [5]. Traditionally, cardiac surgery trainees could only acquire technical skills through direct operating room experience. This apprenticeship model presents several limitations, including restricted training opportunities and potential risks associated with adverse events [6]. However, simulation-based training programs have demonstrated significant efficacy in enhancing technical proficiency, communication skills, and decision-making capabilities in cardiothoracic surgery [7]. Virtual reality (VR) simulators offer distinct advantages over animal models and dry-lab simulators. These advantages include high reproducibility, superior fidelity, unlimited usage potential without physical deterioration, and the ability to pause, replay, and restart scenarios at will, facilitating iterative practice and error analysis [8]. Through the development of personalized, interactive anatomical models, VR simulation provides core technological support across the entire spectrum of smart healthcare applications, including surgical skill training, computer-aided diagnosis, and preoperative planning. Consequently, VR simulation is widely regarded as the most promising solution for robotic surgical skill acquisition [9]. Nevertheless, current virtual reality simulators face several technical challenges, including accuracy in anatomical structure modeling, realism in tissue deformation simulation, authenticity of haptic feedback, and requirements for real-time system performance [10].

To enhance surgical simulation fidelity, various physics-based approaches have been explored for soft-tissue modeling and cutting simulation. The finite element method (FEM) has demonstrated its capability in achieving precise tissue interactions and flexible deformations [11], with frameworks like SOFA providing robust implementation solutions [12]. Position-based dynamics (PBD), on the other hand, offers an alternative approach that balances computational efficiency with physical accuracy [13,14]. Notable implementations include Nvidia FleX and the interactive medical simulation toolkit (iMSTK) [15]. However, traditional organ modeling approaches often treat anatomical structures as isolated entities, overlooking the complex physiological relationships between organs. Such simplified approaches fail to accurately reflect tissue response characteristics in real surgical environments. Therefore, developing a modeling methodology that effectively integrates physiological constraints and captures dynamic coupling relationships between tissues is crucial for improving the simulation accuracy of virtual surgical systems.

Haptic feedback has become a key focus in virtual surgical simulation, enabling surgeons to perceive tactile sensations during instrument–tissue interactions [16,17]. While successfully implemented in systems like the da Vinci Xi, achieving high-quality haptic feedback in virtual simulations faces challenges including real-time computation versus accuracy trade-offs, complex tissue mechanical modeling, and system latency issues affecting feedback synchronization.

Based on these considerations, this study addresses the unique surgical challenges posed by the internal mammary artery’s proximity to the beating heart, where cardiac pulsation transmitted through the thoracic cavity significantly restricts operating space and impacts instrument control. We developed a comprehensive virtual simulation environment for internal mammary artery harvesting, incorporating both anatomical relationships and cardiac motion effects. The system successfully implements various surgical interactions including soft tissue deformation, tissue cutting, and removal operations. This research provides novel insights and practical experience for the further development of virtual surgical simulation systems, particularly in accurately replicating the complex dynamics of thoracic surgical procedures.

## 2. Materials and Methods

**Figure 1:**
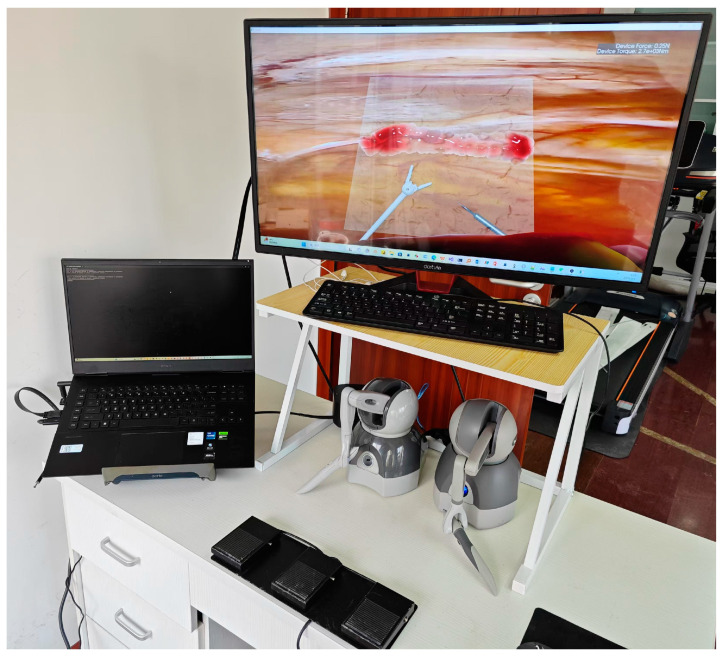
Hardware configuration of the proposed virtual surgical simulation platform. The interface consists of two haptic devices, foot pedals for electrocautery activation, and a high-resolution display for surgical scene visualization.

We propose the design, development, and evaluation of a haptic-enabled virtual simulation platform for robotic IMA harvesting. The system integrates essential hardware components including dual haptic interfaces for bimanual instrument control, foot pedals for electrocautery activation, and high-resolution displays for 3D visualization, as shown in Figure 1. A dedicated software interface enables precise control of virtual robotic instruments with real-time force feedback during tissue manipulation and dissection. The haptic rendering algorithm provides dual-channel force feedback incorporating both mechanical tissue resistance and thermal effects during electrocautery. The simulation environment, developed using iMSTK [15], features detailed anatomical models including the IMA, surrounding connective tissues, and a beating heart model to recreate the challenging surgical field conditions. This comprehensive platform allows surgeons to practice critical technical skills for robotic IMA harvesting in a risk-free virtual environment.

### 2.1. Surgical Anatomy and Motion Constraints

The anatomical layering of the anterior chest wall demonstrates a clear superior-to-deep arrangement, where the superficial fascia forms a membranous layer of connective tissue immediately deep into the dermis, containing collagen, elastic fibers, and neurovascular structures. Deep to this lies the subcutaneous fat, composed of adipose and loose connective tissue with small blood vessels. The IMA is situated much deeper, typically 2–3 cm lateral to the sternum, running vertically behind the costal cartilages and deep to the pectoralis minor muscle. These vessels course between the pleura and internal intercostal muscles within the parasternal region, demonstrating the significant depth at which they reside compared to the more superficial fascial and adipose layers. It is noteworthy that since the internal mammary artery is located on the inner thoracic wall, its anatomical position is adjacent to the constantly beating heart. The periodic motion of the heart is transmitted through the thoracic cavity to the surgical area, significantly limiting the operating space. Particularly during left internal mammary artery harvesting, cardiac pulsation directly affects the precise control of surgical instruments, which is one of the key factors contributing to the technical difficulty of this procedure. Therefore, when constructing virtual simulation scenarios, it is necessary to consider not only the anatomical relationships of various tissue layers but also simulate the impact of cardiac pulsation on the surgical area to more realistically reproduce the characteristics of the surgical environment.

### 2.2. Images and Processing

**Figure 2:**
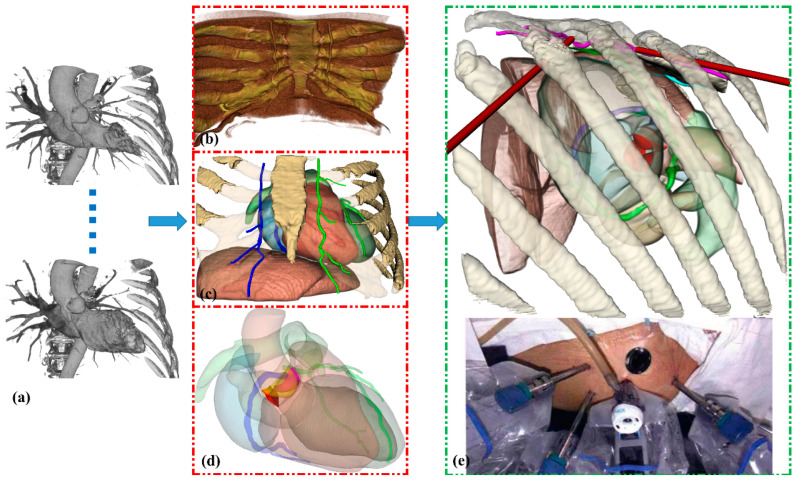
Visualization of key anatomical structures and surgical setup for robotic CABG. (**a**) ECG-gated sequential images. (**b**) Thoracic cage and diaphragm. (**c**) Internal mammary artery. (**d**) Whole-heart mesh incorporating coronary arterial system and aortic valve anatomy. (**e**) Three-dimensional visualization of robotic CABG surgical scene showing optimal port placement configuration, including camera port, instrument ports, and assistant port positions on the left chest wall. Red dashed boxes indicate individual anatomical structures. Green dashed box shows the complete surgical scene.

Cardiac CT imaging was performed using a Revolution Apex scanner (GE Healthcare, Milwaukee, WI, USA) during end-expiratory breath-hold with the patient in normal sinus rhythm. Image reconstruction was conducted across 11 temporal phases of the cardiac cycle, spanning from −5% to 106% of the R-R interval. The acquisition parameters included an isotropic slice thickness and increment of 0.625 mm, with an in-plane spatial resolution of 0.23 × 0.23 mm. The mean effective radiation dose, calculated in accordance with European Guidelines for Quality Criteria for Computed Tomography, was 10.49 mSv. Our methodological approach was implemented using MONAI [18] framework through the Auto3DSeg pipeline. Initial segmentation and reconstruction of the skeletal structures (ribs, vertebrae, sternum), diaphragm, and internal mammary artery were performed on end-diastolic phase (70%) CT images. Subsequently, HeartDeformNet [19] was employed for automated multi-chamber cardiac segmentation, including the left and right ventricles, left and right atria, LV myocardium, aorta, and pulmonary artery across all cardiac phases. Coronary artery segmentation was performed on the 70% phase images using SimVascular-v2023.03.27 [20], where an experienced cardiologist delineated arterial contours on cross-sectional planes perpendicular to the vessel centerlines. The dynamic model was completed by registering nine cardiac phases (10–90%) to the reference 70% phase, deriving transformation matrices for the 3D surface model points of coronary arteries. As shown in Figure 2, these derived transformation matrices enabled the reconstruction of time-resolved (4D) anatomical structures throughout the complete cardiac cycle (Supplementary Materials-Video S1).

### 2.3. Biomechanical Framework for Dynamic Thoracic Wall Simulation

**Figure 3:**
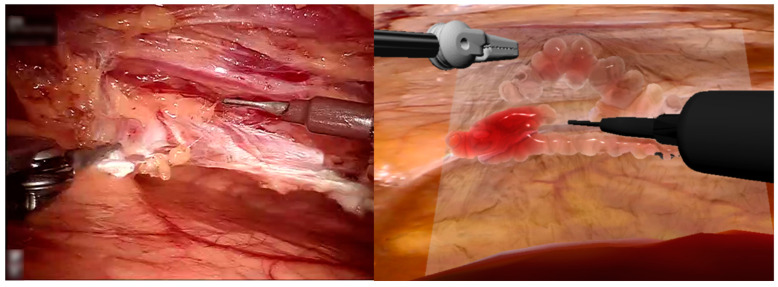
Comparison between real surgical procedure and virtual surgical simulation.

A novel haptic-enabled surgical simulator was developed for robotic-assisted CABG procedures. Figure 3 illustrates the parallel comparison between clinical robotic CABG operations and the virtual surgical environment, highlighting the correlation between real-world surgical manipulation and haptic-rendered simulation scenarios.

**Figure 4:**
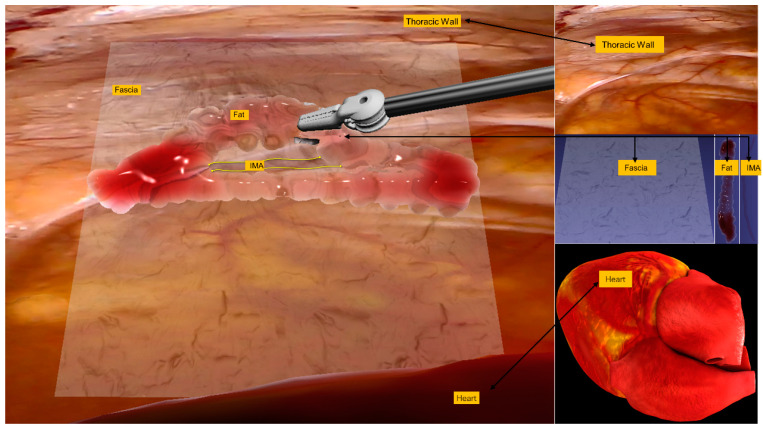
Multi-tissue biomechanical simulation framework of thoracic wall. The framework incorporates four anatomical components (fascia superficialis, IMA, subcutaneous adipose tissue, and beating heart), utilizing multi-resolution mesh discretization and tissue coupling mechanisms for real-time surgical simulation.

To achieve realistic surgical interaction, we present a comprehensive biomechanical simulation framework for thoracic wall tissues, as shown in Figure 4, incorporating four primary anatomical components: fascia superficialis, IMA, subcutaneous adipose tissue, and dynamic heart. The system employs a multi-resolution approach with distance and dihedral angle constraints to model tissue mechanics. The fascia and IMA are simulated using triangular mesh discretization, while the subcutaneous fat is represented through a connective tissue strand network. A bidirectional coupling mechanism enables real-time deformation propagation between tissues, optimized for computational efficiency while maintaining anatomical accuracy. This framework is specifically designed for real-time surgical simulation applications (Supplementary Materials-Video S2).

#### 2.3.1. Fascia Superficialis Simulation

**Figure 5:**
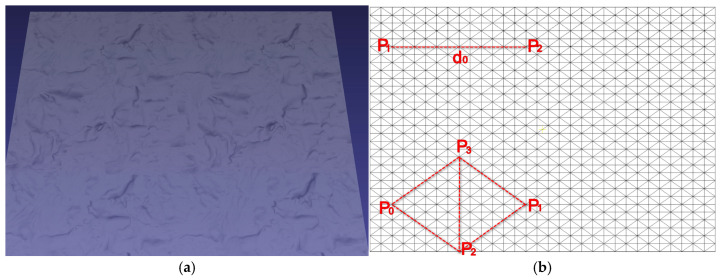
Fascia superficialis discretization and constraint mechanisms. (**a**) Triangular mesh representation of fascia superficialis, where vertices encode tissue surface geometry and faces define the connectivity structure. (**b**) Biomechanical constraints governing tissue deformation: distance preservation constraint maintains structural integrity, while dihedral angle constraint controls bending behavior.

The fascia superficialis simulation framework is based on PBD, employing a triangular mesh discretization M=(V,F) on a rectangular domain $D=\left[0,\omega \right]\times [0,h]$ (Figure 5a), where $V={\left\{v_{i}\right\}}_{i=1}^{n}\subset R^{3}$ represents the set of vertex positions and $F={\left\{f_{j}\right\}}_{j=1}^{m}\subset N^{3}$ denotes the set of triangular faces defining the mesh connectivity. The dynamics are governed by a two-stage solver incorporating position updates and constraint satisfaction. For each vertex i, the position $x_{i}^{*}$ is initially predicted as:

(1) $$x_{i}^{*}=x_{i}\left(t\right)+v_{i}\left(t\right)∆t+\frac{1}{2}a_{ext}{∆t}^{2}$$

where $x_{i}\left(t\right)$ is the current position at time t, $v_{i}\left(t\right)$ denotes the velocity at time t, ∆t is the time step size, $a_{ext}$ represents external acceleration, primarily gravity. The system is constrained by two primary mechanisms reflecting the biomechanical properties of fascia superficialis: distance preservation constraint and dihedral angle constraint (Figure 5b). The tissue’s tensile resistance is modeled through a distance preservation constraint:

(2) $$C_{d}\left(p_{1},p_{2}\right)=\left|p_{1}-p_{2}\right|-d_{0}$$

where $d_{0}$ represents the rest length between particle pairs $p_{1}$ and $p_{2}$. This constraint ensures the maintenance of structural integrity by preserving inter-particle distances during deformation. The dihedral angle constraint $C_{\theta }$ operates on four vertices $p_{0}$, $p_{1}$, $p_{2}$, $p_{3}$ forming adjacent triangles with a shared edge $p_{2}p_{3}$.

(3) $$C_{\theta }\left(p_{1},p_{2},p_{3},p_{4}\right)=\mathrm{atan}2\left(n_{1}\times n_{2}\cdot (p_{3}-p_{2}),\left|p_{3}-p_{2}\right|\cdot n_{1}\cdot n_{2}\right)-\theta_{rest}$$

where atan2(y,x) returns the angle θ∈[−π,π] such that x=rcos⁡θ and y=rsin⁡θ for some r>0. $n_{1}=\frac{\left(p_{2}-p_{0})\times (p_{3}-p_{0}\right)}{\left|\left(p_{2}-p_{0})\times (p_{3}-p_{0}\right)\right|}$, $n_{2}=\frac{\left(p_{3}-p_{1})\times (p_{2}-p_{1}\right)}{\left|\left(p_{3}-p_{1})\times (p_{2}-p_{1}\right)\right|}$, $\theta_{rest}$ denotes the initial dihedral angle measured in the rest configuration of the mesh, serving as the reference state for the bending constraint.

These constraints are iteratively satisfied through position corrections ∆x=λ∇C, where $\lambda =-\frac{C(x)}{{\left|\nabla C(x)\right|}^{2}}$. The system incorporates both linear damping force $F_{d}$ and angular damping force $T_{d}$ to simulate the viscoelastic properties of fascial tissue.

(4) $$\left\{\begin{array}{c} F_{d}=-k_{l}v\;\;\;\;\;\;{\;\;k}_{l}=0.03\;kg/s\\ T_{d}=-k_{a}\omega \;\;\;\;\;\;{\;k}_{a}=0.01\;kg\cdot m^{2}/s \end{array}\right.$$

where v is the linear velocity and ω is the angular velocity of the particles. The numerical integration employs a time step of ∆t=5 ms with 5 solver iterations per step, ensuring stable and anatomically plausible deformation behavior. Boundary conditions are enforced by fixing perimeter vertices, while uniform mass distribution is applied across all vertices. This formulation provides a robust balance between computational efficiency and physiological accuracy, making it particularly suitable for real-time surgical simulation applications involving fascial tissue manipulation.

#### 2.3.2. Multi-Resolution IMA Vessel Simulation

**Figure 6:**
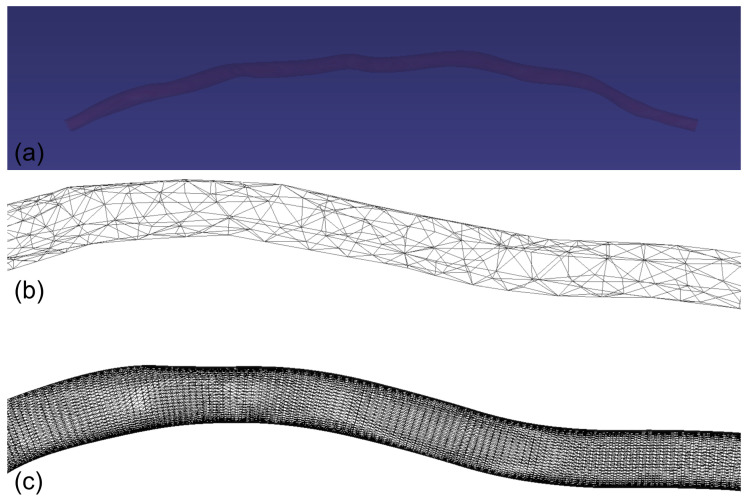
Multi-resolution representation of the Internal Mammary Artery (IMA). (**a**) IMA visualization in the virtual surgical environment demonstrating anatomically accurate vessel positioning and appearance. (**b**) Low-resolution simulation mesh optimized for efficient physical computation. (**c**) High-resolution visualization mesh providing enhanced geometric detail.

Similar to the fascia model, the IMA vessel’s mechanical behavior is governed by a dual-constraint system: a distance preservation constraint with high stiffness coefficient to maintain vessel wall integrity, and a dihedral angle constraint with lower stiffness to model the characteristic bending behavior of vascular structures. The simulation employs a multi-resolution approach, utilizing a low-resolution mesh for physical computations (Figure 6b) and a high-resolution mesh for visualization (Figure 6c), optimizing both computational efficiency and visual fidelity. The system employs uniform mass distribution across all vertices of the simulation mesh and notably operates in a gravity-free environment to simulate the in situ mechanical conditions of the thoracic cavity.

The numerical framework utilizes position updates of the form $x_{i}^{*}=x_{i}\left(t\right)+v_{i}\left(t\right)∆t$ for each vertex i with constraint satisfaction achieved through iterative position corrections, where $x_{i}^{*}$ represents the predicted position of vertex, $x_{i}\left(t\right)$ is the current position at time t, $v_{i}\left(t\right)$ denotes the velocity at time t, ∆t is the time step size. The high-resolution display mesh is subsequently updated through linear interpolation based on the deformed simulation mesh. This formulation provides a biomechanically plausible representation of IMA deformation during surgical manipulation, with particular emphasis on maintaining vessel wall integrity while allowing for natural flexion and torsion. The model’s high-resolution geometric fidelity, combined with its physically based deformation characteristics and computationally efficient multi-resolution architecture, makes it especially suitable for surgical simulation applications involving thoracic vessel manipulation.

#### 2.3.3. Connective Tissue Modeling

To simulate the physical interaction between the IMA and subcutaneous fat tissue, we established a PBD model with connective tissue strands. The system consists of three main components: a low-resolution IMA mesh, a cylindrical support structure representing the thoracic wall, and interconnecting strands. The IMA and support structure are modeled as static PBD objects with uniform mass distribution:

(5) $$m_{v}=\frac{M_{total}}{N_{v}}$$

where $m_{v}$ represents the mass of each vertex, $M_{total}$ is the total mass, and $N_{v}$ is the number of vertices in the mesh. The connective tissue is represented by a network of strands generated between proximal surfaces, governed by the proximity condition: $d_{ij}\leq d_{max}$, where $d_{ij}$ is the distance between surface elements i and j, and $d_{max}$ is either explicitly specified or automatically calculated as the distance between object centers:

(6) $$d_{max}=\left\|C_{A}-C_{B}\right\|$$

**Figure 7:**
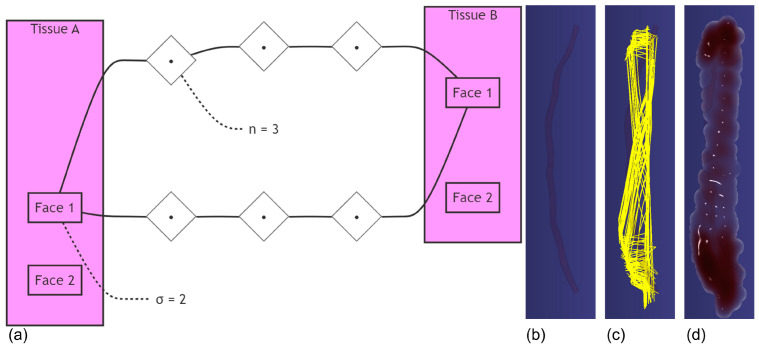
Multi-component system for IMA connective tissue modeling. (**a**) Parameterization of connective tissue strands. The density of connections between tissues is controlled by two key parameters: σ (strands per face) and n (segments per strand). In this example, *σ* = 2 strands emanate from Face 1 of Tissue A, and each strand is subdivided into *n* = 3 segments, creating a network of connection points (•) between the tissues. This parameterization enables precise control over both the density of connections and the granularity of deformation simulation. (**b**) A low-resolution IMA mesh. (**c**) Connective tissue strand network. (**d**) Physically based connective tissue model with SPH.

Subsequently, the total motion disparity $M\left(t\right)$ at each cardiac phase t was computed by integrating the normalized displacement differences across all corresponding EAT–pericardium point pairs within the defined ROI:

(7) $$M\left(t\right)=\sum_{p\in \Omega_{ROI}^{t}}{\left\|{\hat{D}}_{diff}\left(p,q,t\right)\right\|}_{2}$$

where $C_{A}$ and $C_{B}$ represent the centers of the IMA and support structure, respectively. The density of connections is controlled by two parameters: strands per face σ and segments per strand n (Figure 7a). The strands follow the surface normal directions within an allowed angle deviation θ, creating a physically plausible representation of the connective tissue structure (Figure 7c). Each strand is subject to distance constraints and implements SPH-based (Smoothed Particle Hydrodynamics) interactions to simulate the viscoelastic behavior of the biological tissue (Figure 7d). This model provides a balanced approach between computational efficiency and physical accuracy, suitable for surgical simulation applications where real-time performance is crucial.

#### 2.3.4. Bidirectional Coupling of IMA and Adipose Tissue

**Figure 8:**
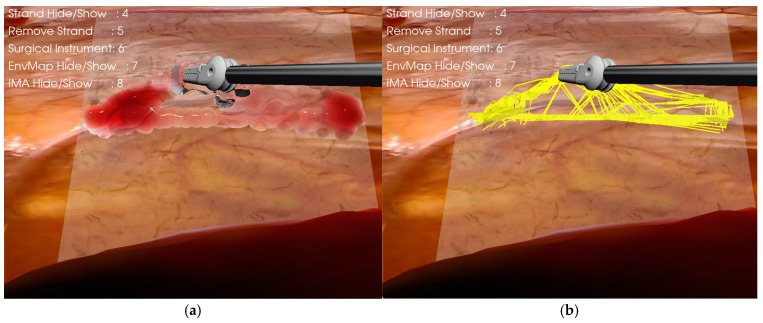
Biomechanical coupling visualization in soft tissue manipulation. (**a**) Visualization of coupled tissue dynamics. Forceps grasping of the fascia superficialis results in concurrent elevation of the adherent subcutaneous fat, demonstrating the biomechanical coupling between tissue layers. (**b**) Deformation mapping mechanism between fascia superficialis and adjacent connective tissue strands.

As shown in Figure 8, we implemented a dynamic coupling mechanism between the IMA and surrounding adipose tissue, utilizing a real-time deformation propagation system. The coupling is achieved through a bidirectional force transfer model that operates on discrete point sets.

The deformation mapping between the IMA and connective tissue strands is governed by:

(8) $$O_{l,i}={\hat{O}}_{ima,j}\cdot min(\left|O_{ima,j}\right|,\lambda_{max})$$

where $O_{l,i}$ is the offset of the *i*-th point in the connective tissue line mesh, $O_{ima,j}$ is the offset of the corresponding *j*-th point in the IMA mesh, ${\hat{O}}_{ima,j}$ represents the normalized direction vector, $\lambda_{max}$ is the maximum allowed deformation magnitude.

For the inverse coupling from adipose tissue to IMA, the deformation transfer is described by:

(9) $$O_{ima,i}=\left\{\begin{array}{c} {\hat{O}}_{fat,j}\cdot \min\left(\left|O_{fat,j}\right|,\lambda_{max}\right)\;\;\;\;\;\mathrm{for}\;\beta =1\\ 0.8\cdot O_{ima,i}\;\;\;\;\;\;\;\;\;\;\;\;\;\;\;\;\;\;\mathrm{for}\;\beta =0 \end{array}\right.$$

where $O_{fat,j}$ represents the offset of the corresponding fat tissue point, and $\beta \in \left\{0,1\right\}$ serves as the coupling state indicator. This bidirectional coupling mechanism ensures physically plausible deformation behavior while maintaining computational efficiency through parallel processing. The damping coefficient of 0.8 in the non-coupled state (β=0) prevents unrealistic oscillations and provides smooth motion decay. To optimize runtime performance, the vertex coupling relationships are pre-computed and stored in mapping arrays, eliminating the need for costly spatial queries during simulation. This approach significantly enhances computational efficiency while maintaining physical accuracy in the deformation propagation process.

### 2.4. Interactive Haptic-Enabled Surgical Manipulation and Cutting

This section presents a comprehensive framework for surgical interaction modeling, encompassing four key components: (1) a topology-preserving cutting method for fascia superficialis simulation using dynamic mesh modification; (2) a geometric intersection-based algorithm for connective tissue cutting within adipose structures; (3) a precise kinematic model for surgical instrument motion control, particularly focusing on forceps manipulation; and (4) a dual-channel haptic feedback system for electrocautery simulation with thermal effects. These components collectively enable realistic surgical tissue manipulation and cutting operations while maintaining computational efficiency for real-time performance.

#### 2.4.1. Topology-Preserving Cutting Method

**Figure 9:**
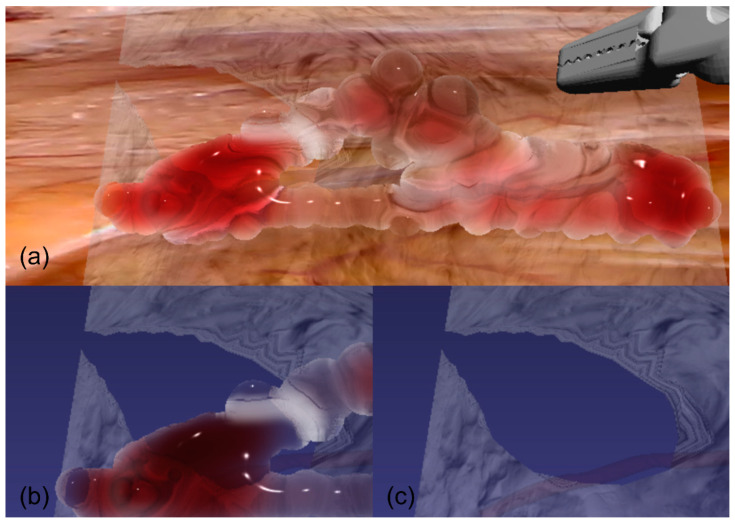
Topological modification scheme for fascia superficialis cutting simulation. (**a**) Complete surgical scene showing the integrated fascia superficialis cutting simulation with all anatomical components. (**b**) Magnified view of the cutting region with chest wall model hidden, highlighting the interaction between fascia superficialis and surrounding tissues. (**c**) Further magnified view with adipose tissue hidden, demonstrating the detailed fascia superficialis structure and cutting dynamics.

As shown in Figure 9, we present a cutting algorithm that implements a dynamic topological modification scheme within the PBD framework, specifically designed for fascia superficialis simulation. Given an input mesh M=(V,E) and a cutting geometry $G_{c}$ with proximity threshold ϵ, where $V={\left\{v_{i}\right\}}_{i=1}^{n}\subset R^{3}$ denotes the set of vertex positions, with n being the total number of vertices. $E={\left\{e_{k}\right\}}_{k=1}^{m}\subset N^{2}$ represents the set of edges, where each edge $e_{k}=(i,j)$ connects vertices $v_{i}$ and $v_{j}$. The algorithm first identifies vertices within the cutting zone by computing signed distances $d_{i}=dist(v_{i},G_{c})$ where $\left|d_{i}\right|<\epsilon$. The mesh undergoes local remeshing to generate new vertices $V_{new}$ and modified connectivity $E_{new}$, resulting in an updated mesh $M_{new}=(V\cup V_{new},E_{new})$. The system maintains constraint coherence through explicit tracking of removed and added constraint sets, while simultaneously propagating both current vertex positions $x_{i}(t)\to x_{i}^{\prime }(t)$ and reference configurations $x_{i}^{0}\to {{(x}_{i}^{0})}^{\prime }$. This approach ensures geometric consistency and physical plausibility while supporting real-time performance through efficient constraint management.

#### 2.4.2. Connective Tissue Surgical Cutting Simulation

**Figure 10:**
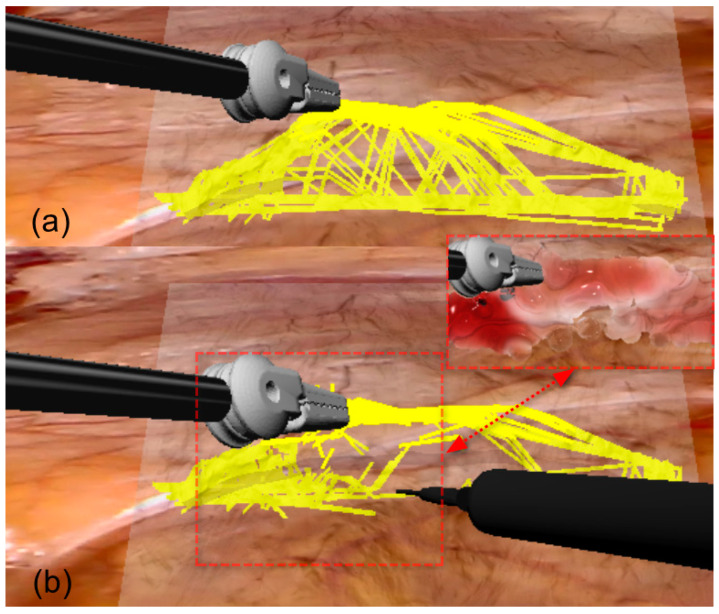
Illustration of our proposed cutting approach for deformable adipose tissue simulation. (**a**) Initial configuration of connective tissue strands before cutting, showing the natural structural arrangement within adipose tissue. (**b**) Post-cutting state, where two cutting tools are separating the tissue, and the yellow connective tissue strands have been cut. The red dashed box marks the cutting area, where changes in tissue structure and the effects after cutting can be observed. Red arrows point to the tissue separation points caused by cutting.

As shown in Figure 10, we propose a novel connective tissue surgical cutting approach that implements a geometric intersection-based removal algorithm for deformable adipose tissue. The cutting plane P is defined as:

(10) $$\left\{x\in R^{3}:n\cdot \left(x-p\right)=0\right\}$$

where p represents the surgical tool’s position vector and n denotes the unit normal vector derived from tool orientation. The tool’s orientation is characterized by an orthonormal basis $\left\{n,l,f\right\}$, with $n=R\cdot {\left(0,1,0\right)}^{T}$, $l=R\cdot {\left(1,0,0\right)}^{T}$, and $f=R\cdot {\left(0,0,1\right)}^{T}$, where R∈SO(3) is the rotation matrix of the surgical tool. The connective tissue structure is discretized as a set of line segments:

(11) $$S={\left\{s_{i}\right\}}_{i=1}^{N}$$

where each segment $s_{i}=\left\{\left(1-t\right)v_{i}^{0}+tv_{i}^{1}:t\in [0,1]\right\}$ is defined by endpoint vertices $v_{i}^{0}$, $v_{i}^{1}\in R^{3}$. A fascial segment is marked for removal when it satisfies:

(12) $$\exists x\in s_{i}:\left|l\cdot \left(x-p\right)\right|<\frac{\omega }{2}⋀\left|f\cdot \left(x-p\right)\right|<\frac{\omega }{2}⋀n\cdot \left(x-p\right)=0$$

where ω represents the cutting width. The visual representation of the connective tissue undergoes a continuous transformation following

(13) $$d_{t+1}=0.96d_{t}$$

where $d_{t}$ represents the particle diameter at time t, terminating visibility when $d_{t}<6\times 10^{-4}$ to simulate the gradual dissection of fascial layers. This methodology, implemented through PBD with parallel processing and SPH visualization, provides a computationally efficient approach for connective tissue dissection simulation while maintaining physical plausibility and visual fidelity, demonstrating robust handling of topological changes and real-time performance suitable for surgical training applications.

#### 2.4.3. Kinematic Modeling of Surgical Instrument Motion

**Figure 11:**
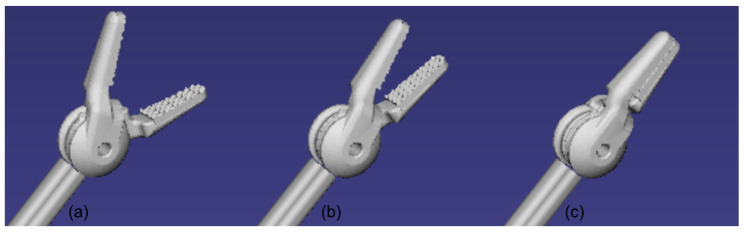
Progressive closure sequence of a DeBakey forceps. (**a**) Maximum opening angle ($30^{{}^{\circ}}$). (**b**) Half-closed position ($15^{{}^{\circ}}$). (**c**) Complete closure (0°).

As shown in Figure 11, the surgical instrument can be mathematically modeled as a kinematic chain operating in the configuration space:

(14) $$Q=SE(3)\times {\left[-\theta_{max},\theta_{max}\right]}^{2}$$

where $SE\left(3\right)$ represents the Special Euclidean group in 3D space, describing the 6-DOF pose of the tool. $\theta_{max}$ is the maximum jaw opening angle, typically set to $30^{{}^{\circ}}$ (Figure 11a). The tool’s world position is governed by the transform hierarchy:

(15) $$T_{world}=T_{controller}\cdot T_{local}\cdot T_{initial}$$

where $T_{controller}$ is the controller’s transformation matrix, $T_{local}$ is the local joint transformation matrix, $T_{initial}$ is the initial offset transformation matrix. The jaw motion follows a time-dependent angular control model:

(16) $$\theta \left(t+dt\right)=\theta \left(t\right)+\dot{\theta }\cdot dt$$

where $\theta \left(t\right)$ is the jaw angle at time t, $\dot{\theta }$ is the angular velocity determined by button inputs, dt is the time step. The upper and lower jaws rotate symmetrically around axis $\vec{\alpha }$ according to: $T_{upper}=R(\theta ,\vec{\alpha })$, $T_{lower}=R(-\theta ,\vec{\alpha })$, where $\vec{\alpha }$ is the rotation axis vector of the jaw hinge. The system transitions between open and closed states based on a threshold angle of $2.86^{{}^{\circ}}$, considering closed when below this value. Both visual and collision geometries are updated through the transform chain. This framework ensures precise control and realistic simulation of the surgical tool’s kinematics during minimally invasive procedures.

#### 2.4.4. Force Feedback Modeling for Electrosurgical Simulation

**Figure 12:**
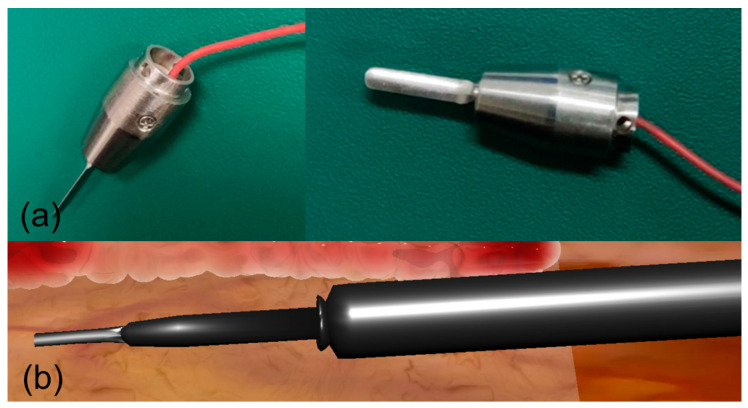
Physical and virtual electrocautery tool for robotic cardiac surgery. (**a**) Dual-view photographs of the actual electrocautery instrument. (**b**) Three-dimensional virtual model of the electrocautery tool.

As a critical component of our robotic CABG simulation platform, we implemented a haptic-enabled electrocautery module (Figure 12) that integrates real-time force feedback rendering with tissue deformation modeling. This module employs a sophisticated dual-channel force feedback algorithm that decomposes tool–tissue interaction forces into two components: the primary channel models perpendicular tissue resistance force

(17) $$F_{n}=k_{d}*d+c_{d}*v_{n},\;{0\leq F}_{n}\leq 5N$$

where $k_{d}$ represents tissue stiffness coefficient, d denotes penetration depth, $c_{d}$ is the damping coefficient, and $v_{n}$ indicates normal velocity vector, while the secondary channel simulates lateral cutting resistance through

(18) $$F_{t}=\mu *F_{n}+k_{c}*v_{t},\;{0\leq F}_{t}\leq 2N$$

where μ represents dynamic friction coefficient, $k_{c}$ denotes cutting resistance coefficient, and $v_{t}$ indicates tangential velocity vector. The total force feedback $F_{total}=F_{n}+F_{t}+F_{thermal}$ incorporates additional thermal modulation based on power settings and tissue properties. The thermal force component $F_{thermal}$ represents a force feedback modulation term that simulates tissue response changes due to electrosurgical thermal effects. It is mathematically expressed as

(19) $$F_{thermal}=k_{t}*P*e^{-\alpha d}$$

where $k_{t}$ is the thermal coefficient specific to tissue type, P represents the electrosurgical power setting (typically 30–50 W), α is the thermal decay coefficient, and d denotes the thermal effect depth. This force component captures several critical aspects of electrosurgical procedures: tissue carbonization resistance changes, cutting/coagulation effects at different power settings, mechanical property alterations due to thermal diffusion, and tissue stiffness variations caused by moisture evaporation. Real-time tissue response is computed using a multi-resolution mesh model, incorporating both elastic deformation and thermal diffusion properties. The force profile varies according to different tissue layers and operational modes (cutting vs. coagulation), with haptic rendering updated at 1000 Hz to ensure stable force feedback, while visual feedback maintains a minimum refresh rate of 60 Hz. This comprehensive electrocautery simulation module enables surgical trainees to practice specific electrocautery techniques with realistic tactile sensation and visual feedback, accurately representing various surgical maneuvers including initial tissue puncture, depth-dependent cutting resistance, and layer-specific mechanical properties.

## 3. Results

The experiment was designed to evaluate the impact of cardiac motion on surgical performance during IMA harvesting using our virtual simulation platform. The simulation scenarios were configured with identical anatomical structures but different cardiac motion states: one with realistic cardiac motion (dynamic group) and one with a static heart (static group).

A standardized surgical task was defined as harvesting a 10 cm segment of the left IMA using virtual robotic instruments. The surgical workflow followed clinical protocols, including initial exposure, vessel identification, and careful dissection along the IMA course. The virtual surgical instruments were modeled based on the da Vinci surgical system’s EndoWrist instruments. A single experienced operator (>100 cases of CABG) performed 10 repetitions for each scenario in randomized order to minimize learning effects. A mandatory 15 min rest period was implemented between trials to reduce operator fatigue. Before formal trials, the operator underwent a standardized training session to achieve stable performance in the virtual environment. All experiments were conducted using the same hardware setup: a workstation equipped with an NVIDIA RTX 4080GPU (NVIDIA Corporation, Santa Clara, CA, USA), Intel i7-13700HK CPU (Intel Corporation, Santa Clara, CA, USA), and 32GB RAM, ensuring consistent computational performance throughout the trials.

Here we propose a novel Spatial Asymmetry Index (SAI) to quantitatively evaluate the spatial distribution characteristics of surgical tool trajectories during IMA harvesting. The SAI is mathematically defined as:

(20) $$SAI=\frac{1}{n}\sum_{i=1}^{n}\frac{d_{i}}{R}\cdot sign(d_{i})$$

where $d_{i}$ represents the perpendicular distance from the tool position to the IMA centerline, R denotes a standardized reference distance (2 mm safety zone radius), and $sign(d_{i})$ indicates the directional component (negative for cardiac side, positive for non-cardiac side), and n represents the total number of sampling points recorded at 60 Hz during the procedure. This metric effectively captures the spatial bias in surgical manipulation, with positive SAI values indicating a preference for operating on the side away from the heart, while values approaching zero suggest more symmetric distribution.

**Figure 13:**
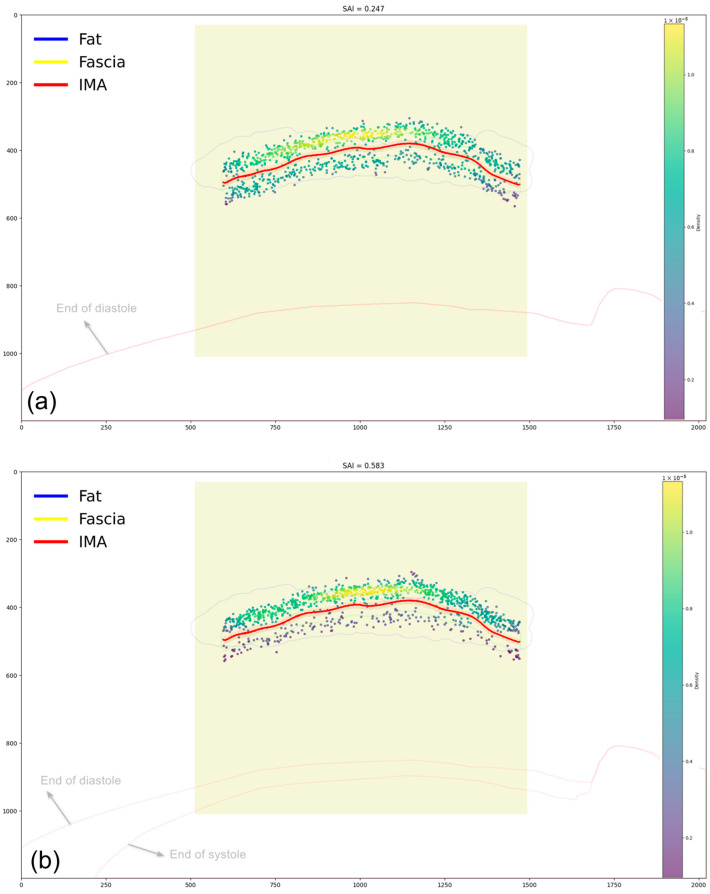
Representative images showing tool position distributions during IMA dissection. (**a**) Static scenario (SAI = 0.247) shows balanced but slightly asymmetric distribution of tool positions along the IMA centerline. (**b**) Dynamic scenario (SAI = 0.583) demonstrates distinct clustering of tool positions on the non-cardiac side. Each heatmap incorporates anatomical structures, IMA centerline trajectory (red), and a density-based color gradient representing the frequency of tool positions. The cardiac contours are projected onto the 2D visualization plane, with red arrows indicating the end-diastolic contour in the static scenario (**a**) and both end-diastolic and end-systolic contours in the dynamic scenario (**b**).

As shown in Table 1, statistical analysis revealed significantly higher SAI values in the dynamic group compared to the static group (0.589 ± 0.029 vs. 0.248 ± 0.024, *p* < 0.001), demonstrating that surgeons systematically adjusted their manipulation strategies to compensate for cardiac motion by maintaining safer working distances from the beating heart. This behavioral adaptation was further validated through spatial density heat maps, which showed distinct clustering of tool positions on the non-cardiac side in the dynamic group (SAI consistently above 0.5), whereas the static group exhibited a more balanced but still slightly asymmetric distribution along the IMA centerline (SAI values around 0.25). As illustrated in Figure 13, the 3D anatomical structures (Fascia, subcutaneous fat tissue, and IMA) and the corresponding tool trajectory data during IMA dissection were projected orthogonally onto a 2D visualization plane for comparative analysis. The SAI thus provides a robust quantitative measure for assessing how cardiac motion influences surgical approach and spatial decision-making during robotic IMA harvesting procedures, with the substantial difference in SAI values (mean difference = 0.341) indicating a clear adaptation of surgical strategy in response to cardiac motion. Additional images from both static and dynamic groups are available in the article/Supplementary Material.

**Figure 14:**
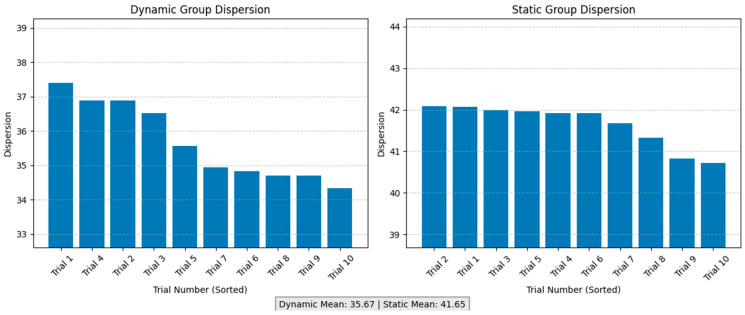
Comparison of surgical tool movement dispersion between dynamic and static groups. The histograms illustrate the dispersion of tool movements across trials in both experimental conditions. The dynamic group demonstrated significantly lower dispersion than the static group (dynamic mean: 35.20, static mean: 41.44), indicating more precise and concentrated tool manipulation under dynamic conditions. The dispersion range for the dynamic group was 33.7–37.3, while the static group showed values between 40.4 and 42.3. This difference suggests that dynamic visual adjustments may help reduce unnecessary tool movements and enhance operational precision. All trials consistently demonstrated that tool control was more stable under dynamic conditions, which has important implications for precision in minimally invasive surgical procedures.

To quantify the spatial distribution of surgical tool positions relative to the IMA centerline, we implemented a statistical dispersion measure based on the standard deviation of vertical distances. For each trial, 1000 points were sampled along the IMA centerline, and corresponding tool positions were generated using a bimodal distribution weighted by the SAI value. The dispersion D was calculated as:

(21) $$D=\frac{1}{\sqrt{N-1}}\sqrt{\sum_{i=1}^{N}{\left(y_{i}-y_{base,i}\right)}^{2}}$$

where N is the number of points (1000), $y_{i}$ represents the *y*-coordinate of the *i*-th tool position, and $y_{base,i}$ is the *y*-coordinate of the *i*-th point on the IMA centerline. This standard deviation-based metric provides a quantitative measure of the spatial spread of surgical tool positions, with larger values indicating greater deviation from the IMA centerline, enabling statistical comparison of spatial variability between static and dynamic surgical conditions. Figure 14 shows decreasing dispersion values across successive trials, suggesting improved operator proficiency and instrument control over time.

## 4. Discussion

This study presents a comprehensive virtual simulation framework for robotic internal mammary artery harvesting, demonstrating several significant advances in surgical simulation technology. Our organ active structure modeling method, incorporating functional relationship constraints, demonstrated high anatomical fidelity with sub-millimeter geometric accuracy. The bidirectional coupling mechanism successfully synchronized multiple anatomical structures, including the internal mammary artery, fascia, adipose tissue, and connective tissue, maintaining temporal consistency within δt≤15ms.

The topology-preserving cutting algorithm for fascia superficialis demonstrated robust performance in maintaining mesh integrity during dissection, while the geometric intersection-based algorithm effectively simulated adipose tissue dissection. Notably, our dual-channel haptic feedback system achieved a 1 kHz update rate, essential for stable force rendering, while incorporating thermal effects in electrocautery simulation. The system maintained consistent performance metrics, including a visual feedback refresh rate of 60 Hz (SD ± 2.5 Hz), ensuring smooth surgical interaction.

Quantitative analysis using the SAI revealed significant behavioral adaptations in surgical technique due to cardiac motion. The dynamic cardiac group demonstrated significantly higher SAI values compared to the static group (0.589 ± 0.029 vs. 0.248 ± 0.024, *p* < 0.001), indicating systematic adjustment of surgical approach to compensate for cardiac motion. Spatial density analysis further validated this finding, showing a distinct clustering of tool positions on the non-cardiac side in the dynamic group.

## 5. Conclusions

This study presents a novel virtual simulation platform for robotic internal mammary artery harvesting that successfully integrates dynamic anatomical modeling with real-time haptic feedback. By incorporating cardiac motion effects and implementing comprehensive tissue interaction mechanisms, our platform provides a realistic training environment that closely mimics the challenges of actual surgical procedures. The quantitative evaluation demonstrates the system’s effectiveness in capturing the essential technical aspects of IMA harvesting, suggesting its potential value as a training tool for robotic cardiac surgery.

### Limitations and Future Research

Several limitations of the current system warrant consideration. The model does not fully account for anatomical variations between patients, and the force feedback models require further clinical validation. Additionally, the simulation environment lacks certain surgical complications, such as bleeding events, and system evaluation is based on data from a single expert operator.

To address these limitations and advance the platform, our future research will leverage the dual haptic device configuration for systematic pairwise comparison studies. This unique setup enables direct comparative assessment methodologies that are particularly valuable for surgical simulation research. We plan to implement studies where users can simultaneously experience different simulation conditions through each haptic device, allowing for immediate comparative evaluation of: (1) alternative haptic rendering algorithms to determine which provides more realistic tissue sensations; (2) varied force scaling parameters to optimize haptic feedback for surgical precision; (3) different tissue deformation models to better represent the mechanical properties of the internal mammary artery and surrounding tissues; and (4) various collision detection techniques to balance computational efficiency with interaction accuracy.

Furthermore, we intend to develop a comprehensive rating scale assessment methodology similar to Koczkodaj et al. [21] to evaluate the proposed artery harvesting simulation in practice. This multi-criteria evaluation approach would allow for systematic assessment of various simulation aspects through standardized rating scales, with the potential for subsequent statistical optimization to identify the most predictive evaluation criteria. Such an approach would not only provide more structured feedback from surgeons but could also help identify which specific simulation elements contribute most significantly to training effectiveness, allowing for more targeted system improvements. The pairwise comparison capabilities of our dual haptic setup would be particularly valuable in this context, enabling direct comparative evaluations of different simulation configurations against established assessment criteria.

While further validation and refinement are needed, this work establishes a promising foundation for the development of next-generation surgical simulation systems.

## Figures and Tables

**Table 1:** Comparison of SAI between dynamic and static groups.

Trial No.	Dynamic Group SAI	Static Group SAI
1	0.583	0.247
2	0.621	0.268
3	0.545	0.221
4	0.602	0.285
5	0.568	0.238
6	0.634	0.276
7	0.592	0.212
8	0.551	0.257
9	0.615	0.228
10	0.577	0.243
Mean ± SD	0.589 ± 0.029	0.248 ± 0.024

## Data Availability

The original contributions presented in this study are included in the article/Supplementary Material. Further inquiries can be directed to the corresponding author(s).

## Supplementary Materials

The following supporting information can be downloaded at: https://www.mdpi.com/article/10.3390/bioengineering12030285/s1, Video S1: Surgical scene showing optimal port placement configuration; Video S2: Multi-tissue simulation of chest wall.
