# Supplementary figures and images for: Dynamic Virtual Simulation with Real-Time Haptic Feedback for Robotic Internal Mammary Artery Harvesting
Source: Bioengineering (Basel). 2025 Mar 13;12(3):285. doi: 10.3390/bioengineering12030285 (PMC11939391; doi:10.3390/bioengineering12030285)

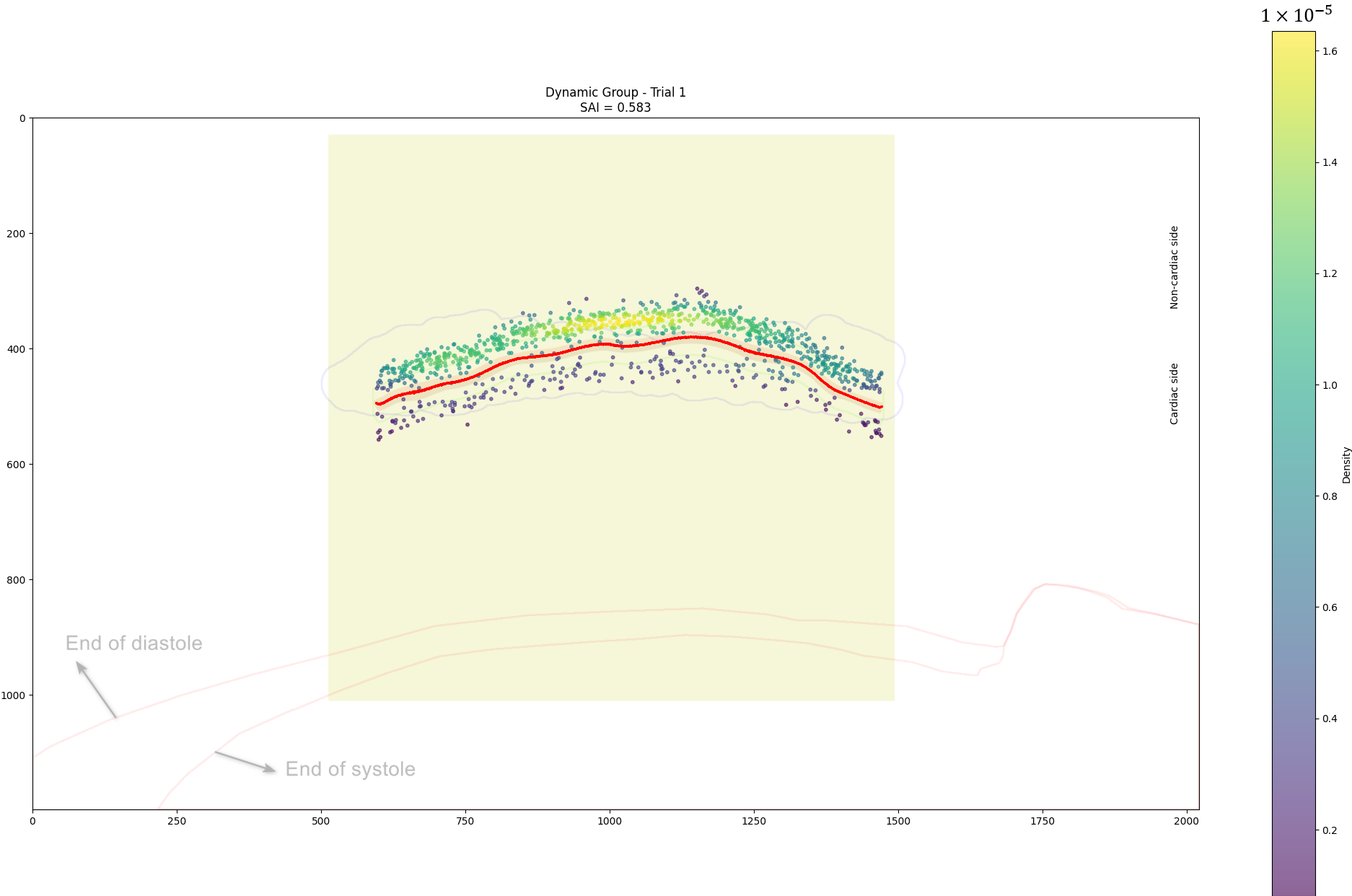

Supplement: Supplementary file 1 [file bioengineering-12-00285-s001.zip › Static and Dynamic groups/dynamic/dynamic_trial_1.png]

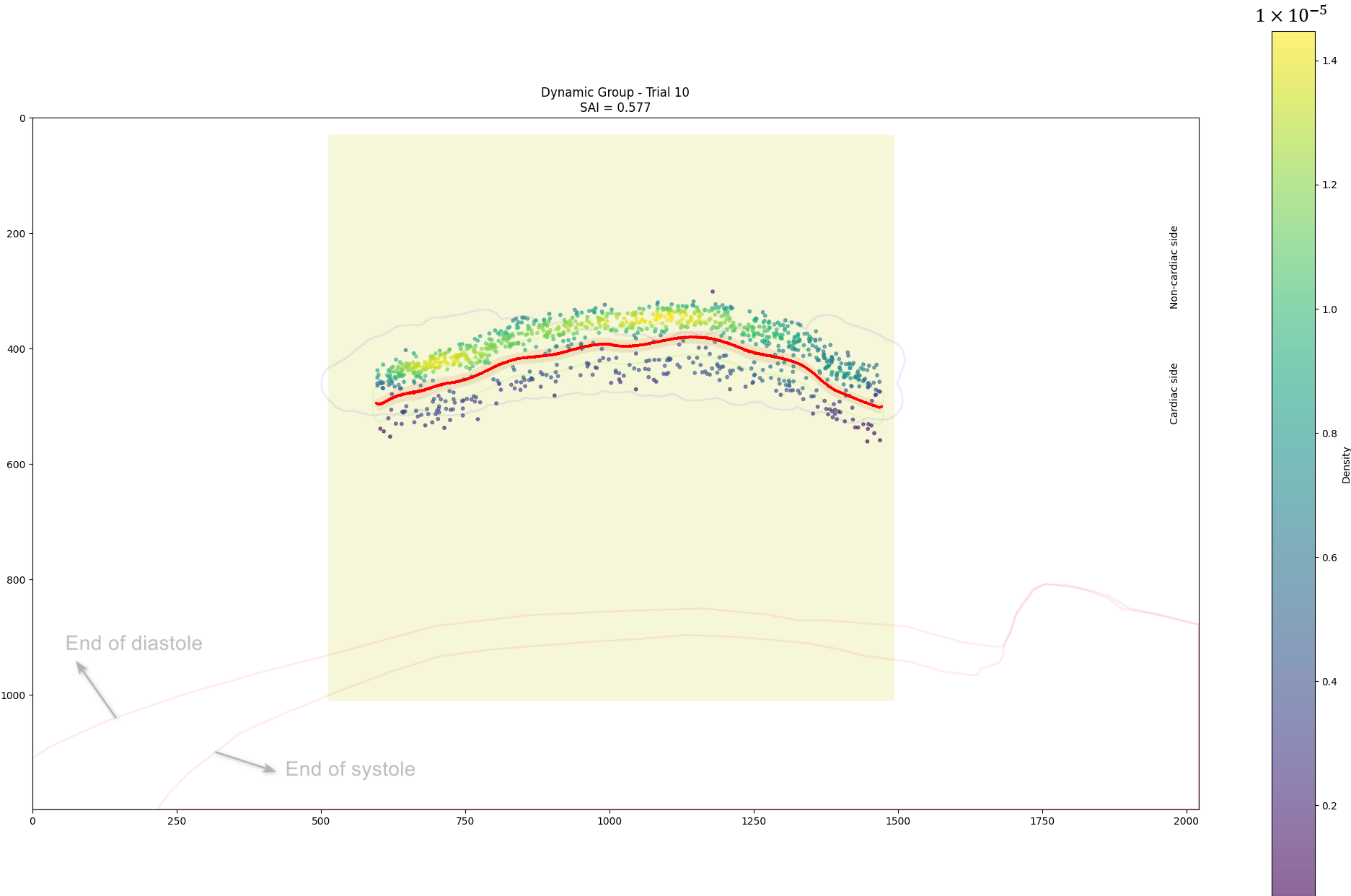

Supplement: Supplementary file 1 [file bioengineering-12-00285-s001.zip › Static and Dynamic groups/dynamic/dynamic_trial_10.png]

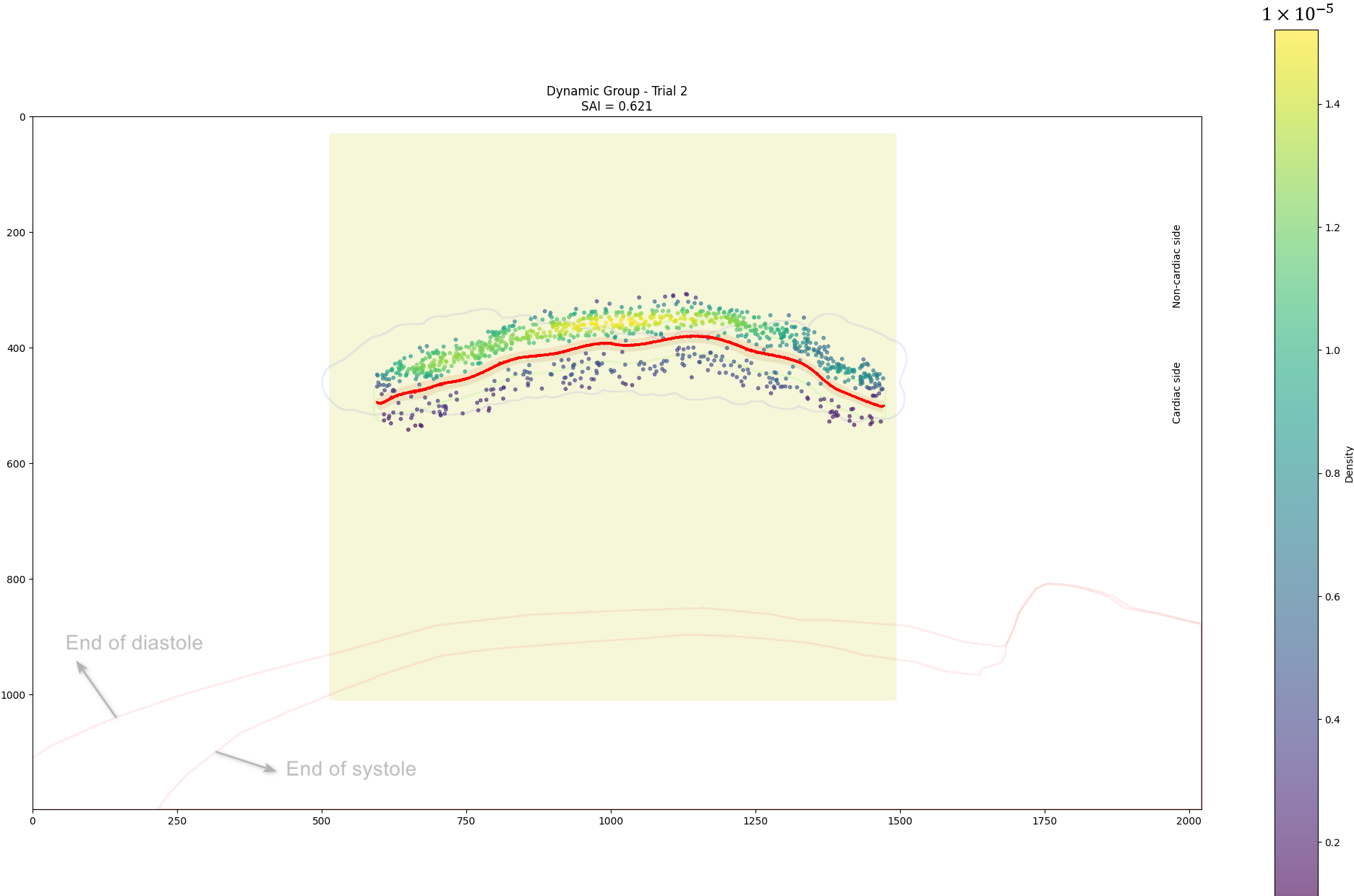

Supplement: Supplementary file 1 [file bioengineering-12-00285-s001.zip › Static and Dynamic groups/dynamic/dynamic_trial_2.png]

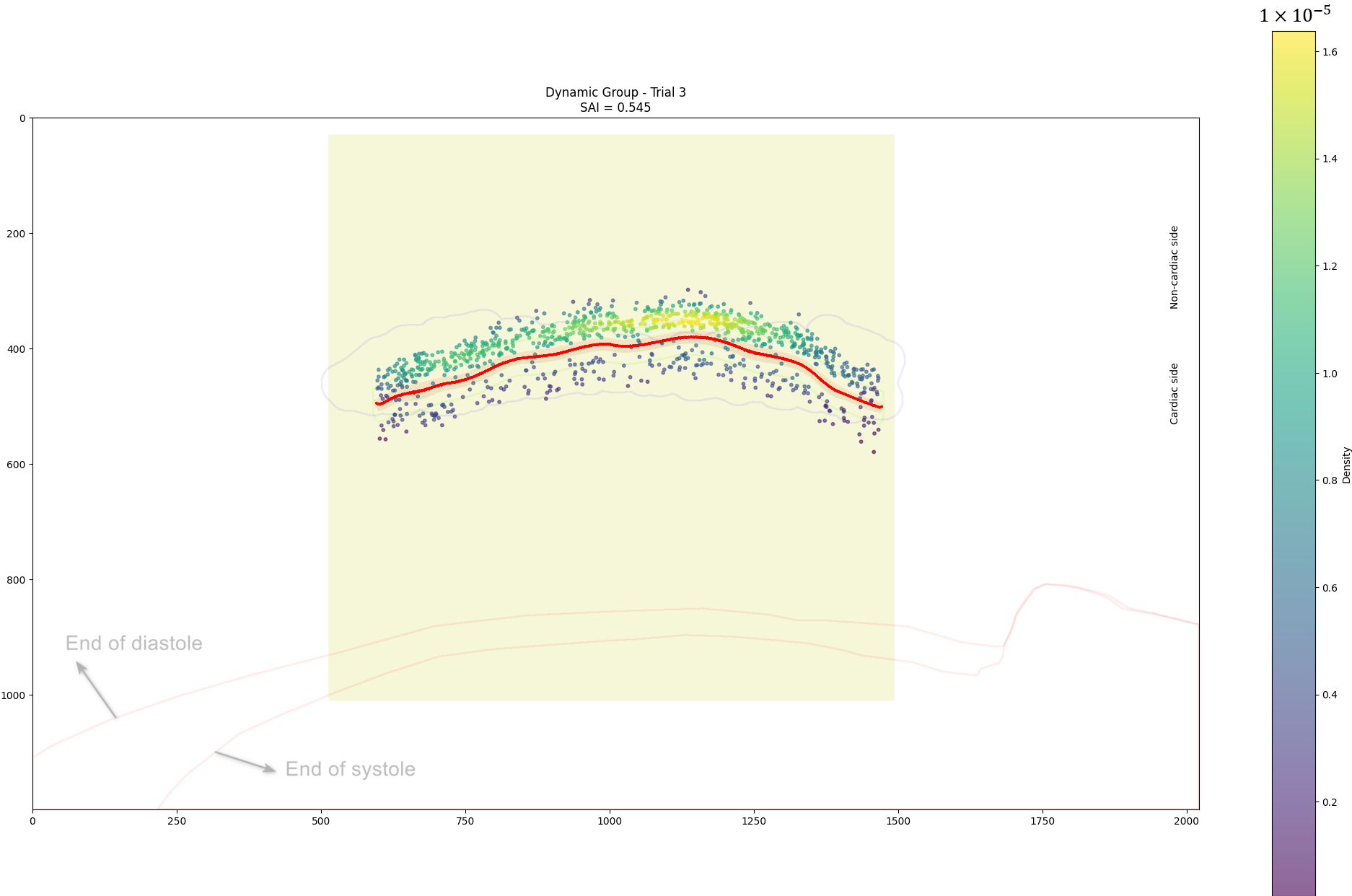

Supplement: Supplementary file 1 [file bioengineering-12-00285-s001.zip › Static and Dynamic groups/dynamic/dynamic_trial_3.png]

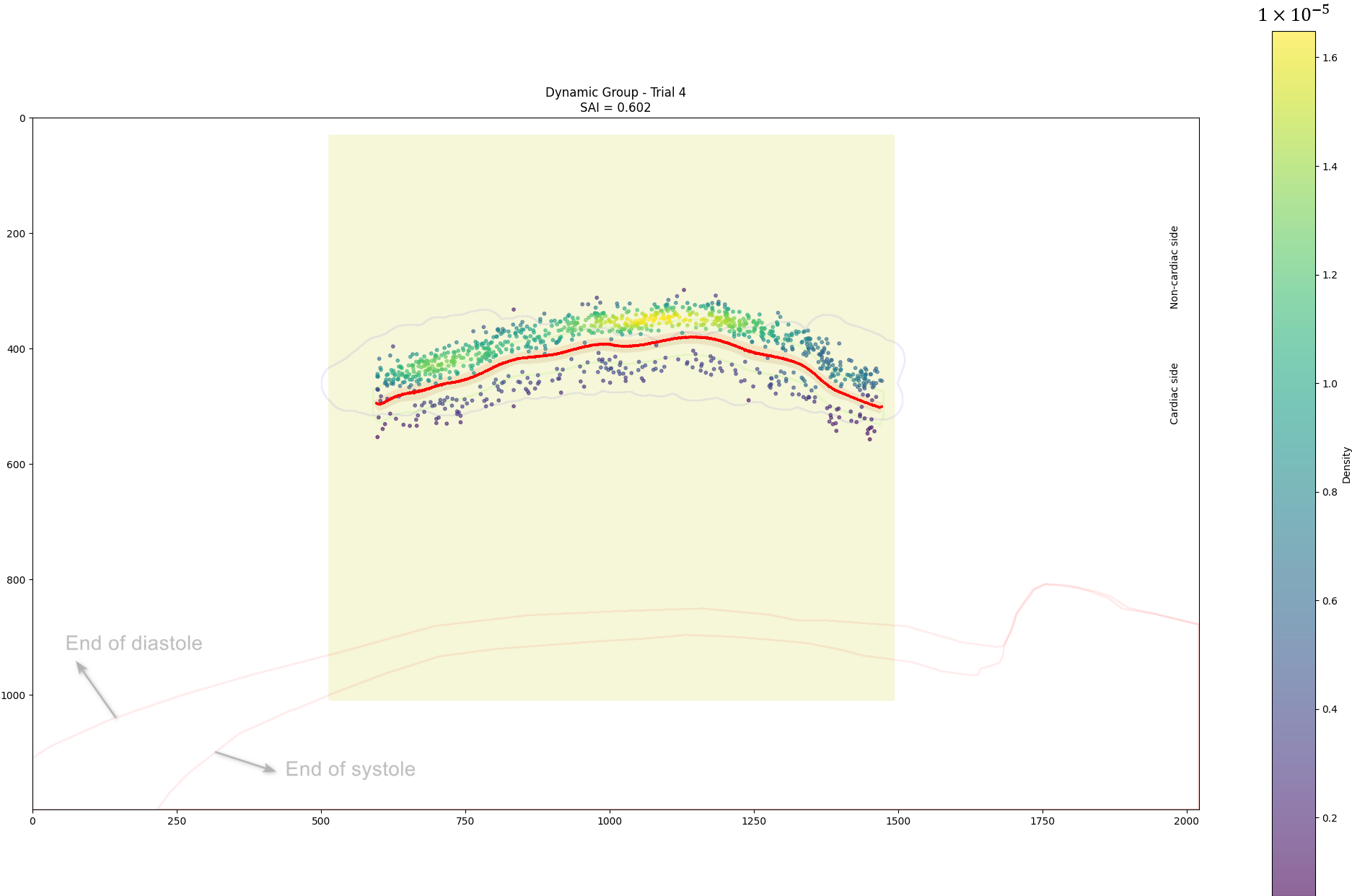

Supplement: Supplementary file 1 [file bioengineering-12-00285-s001.zip › Static and Dynamic groups/dynamic/dynamic_trial_4.png]

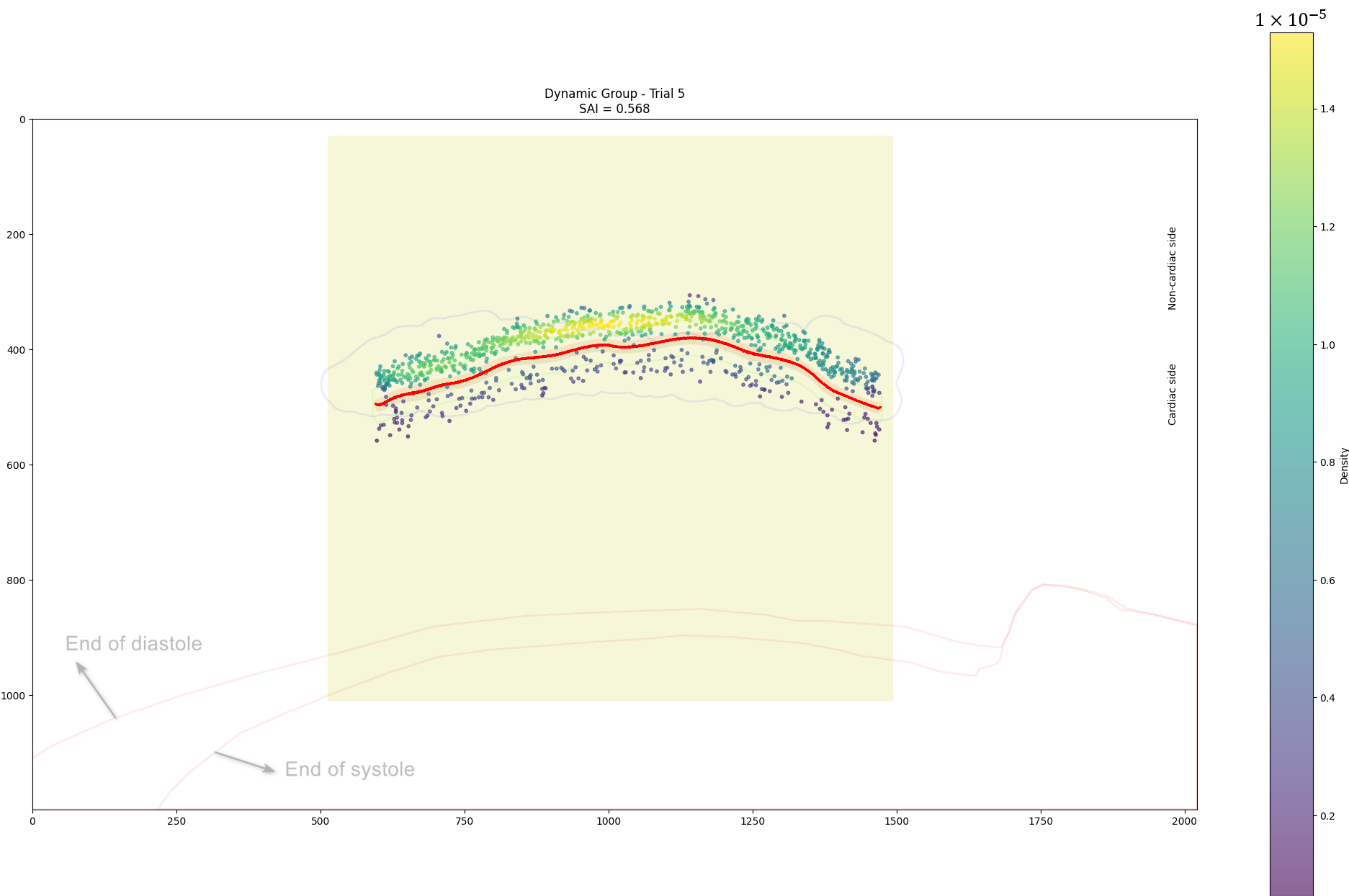

Supplement: Supplementary file 1 [file bioengineering-12-00285-s001.zip › Static and Dynamic groups/dynamic/dynamic_trial_5.png]

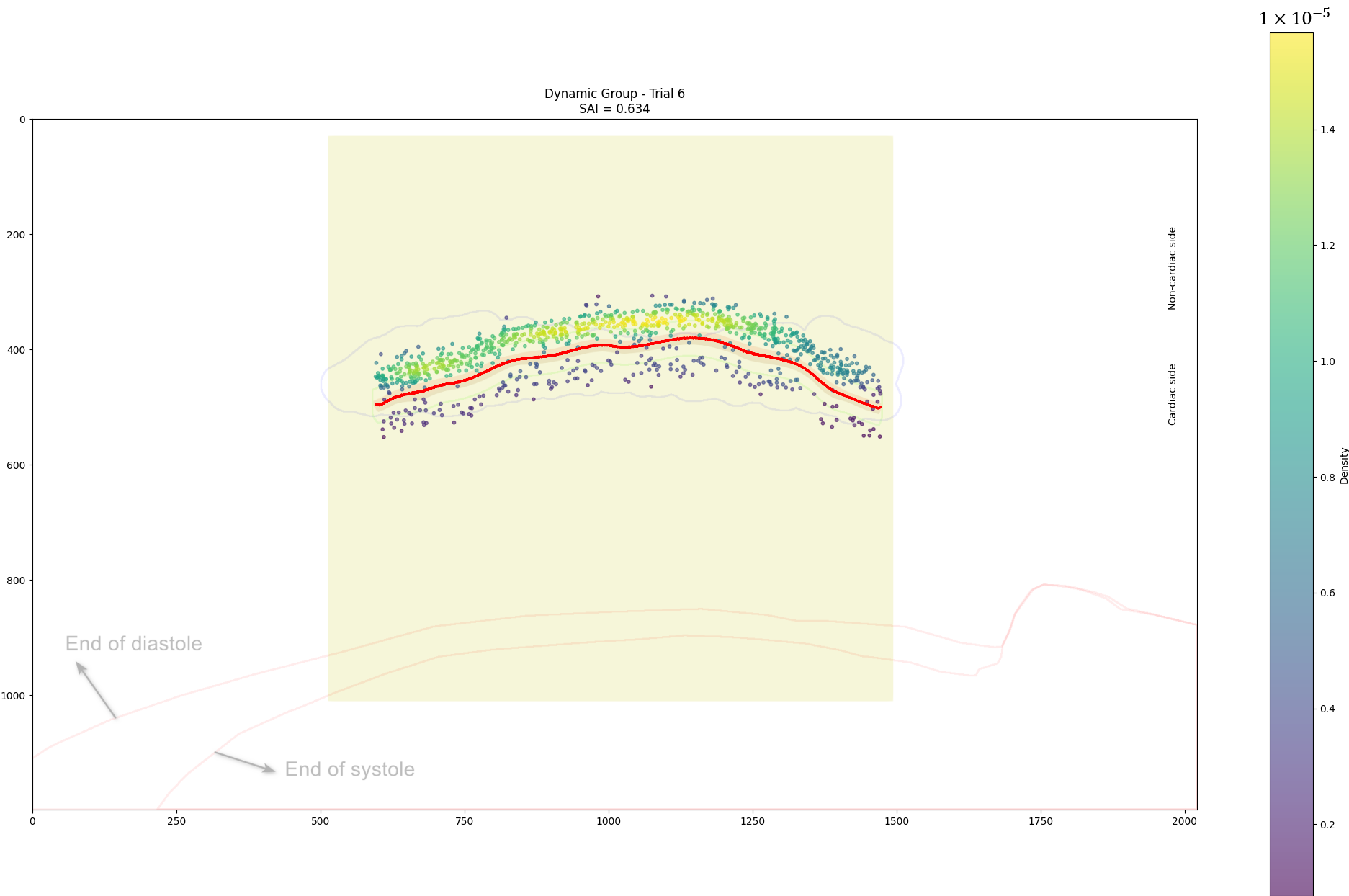

Supplement: Supplementary file 1 [file bioengineering-12-00285-s001.zip › Static and Dynamic groups/dynamic/dynamic_trial_6.png]

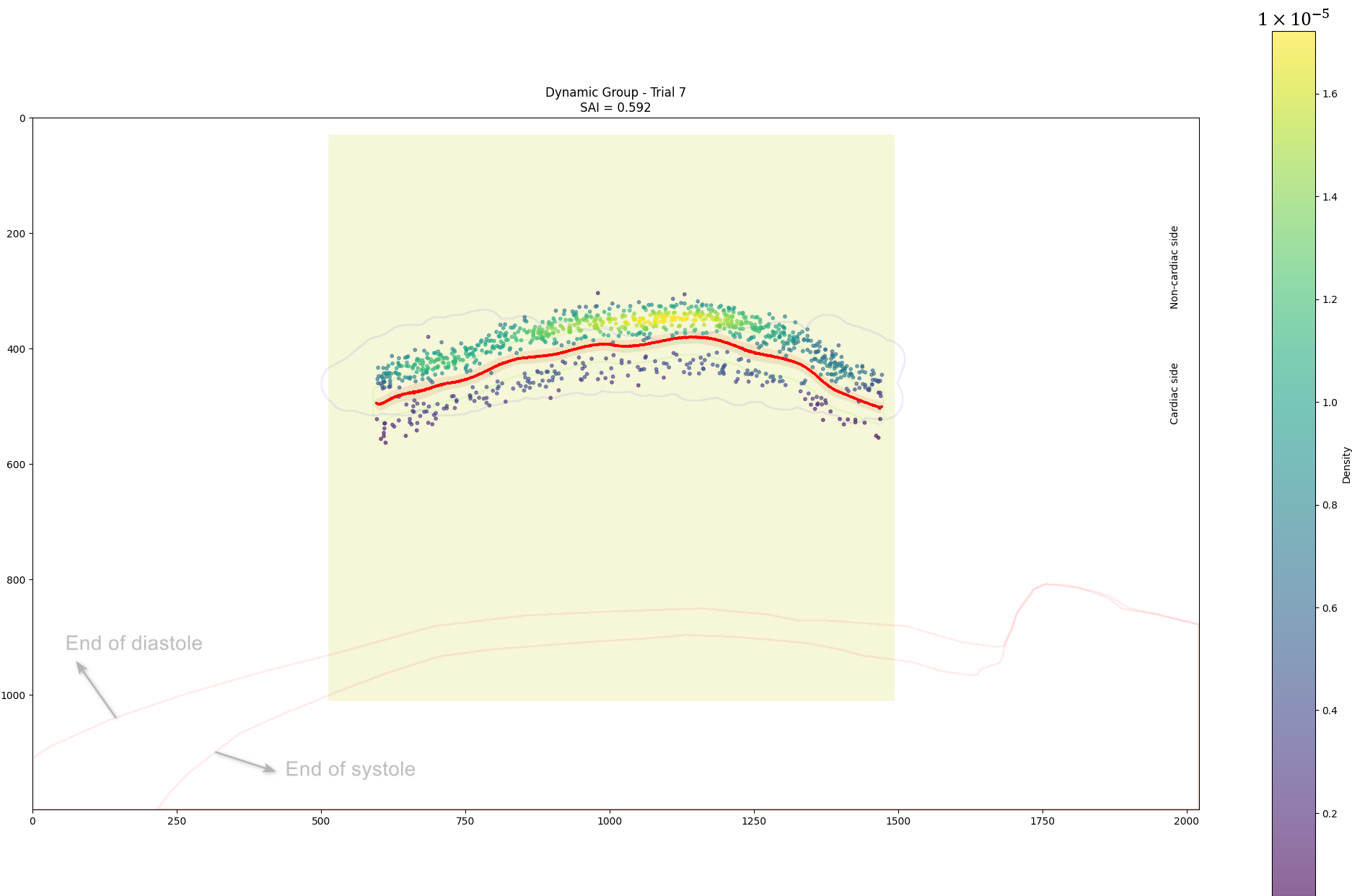

Supplement: Supplementary file 1 [file bioengineering-12-00285-s001.zip › Static and Dynamic groups/dynamic/dynamic_trial_7.png]

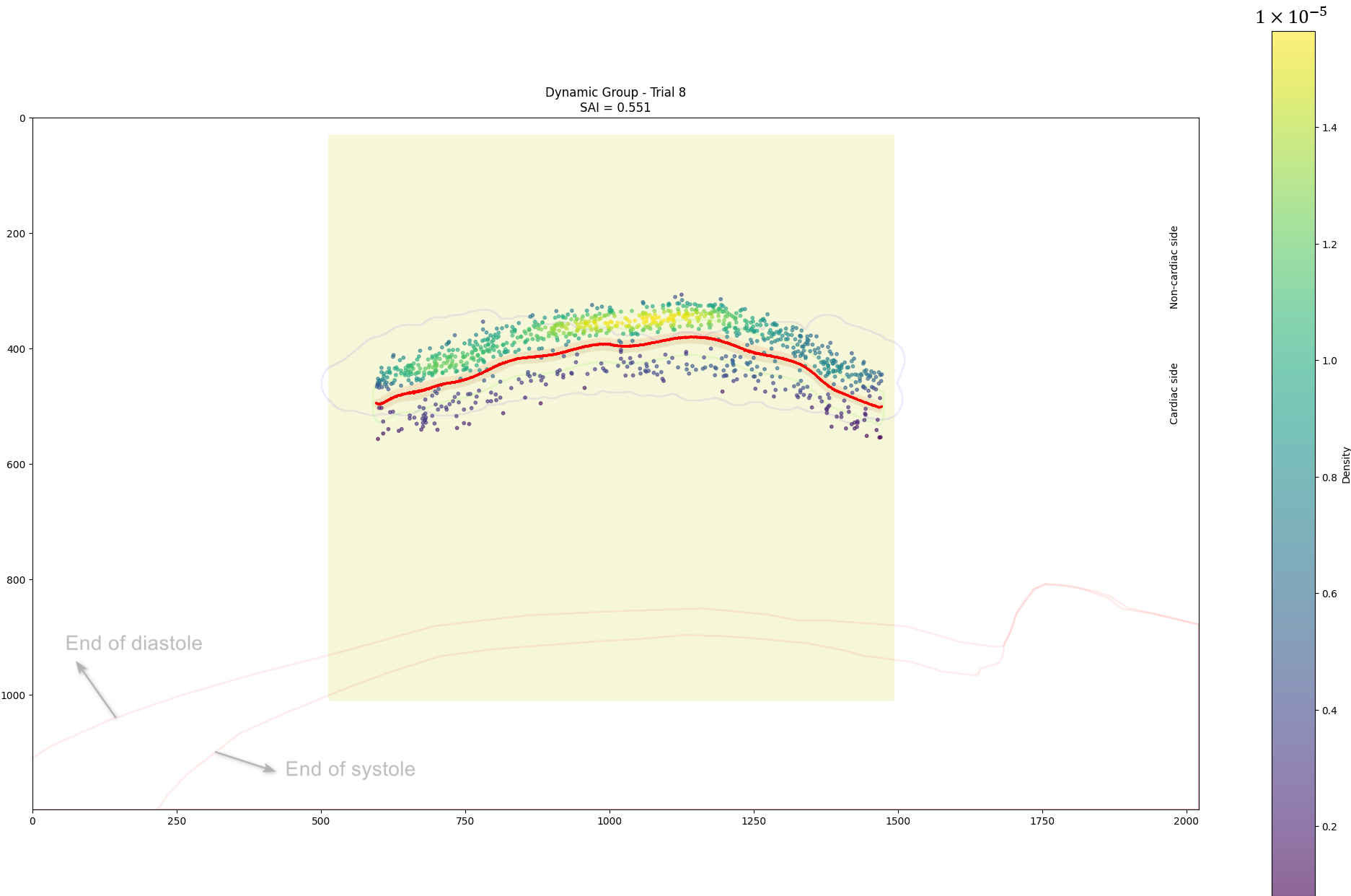

Supplement: Supplementary file 1 [file bioengineering-12-00285-s001.zip › Static and Dynamic groups/dynamic/dynamic_trial_8.png]

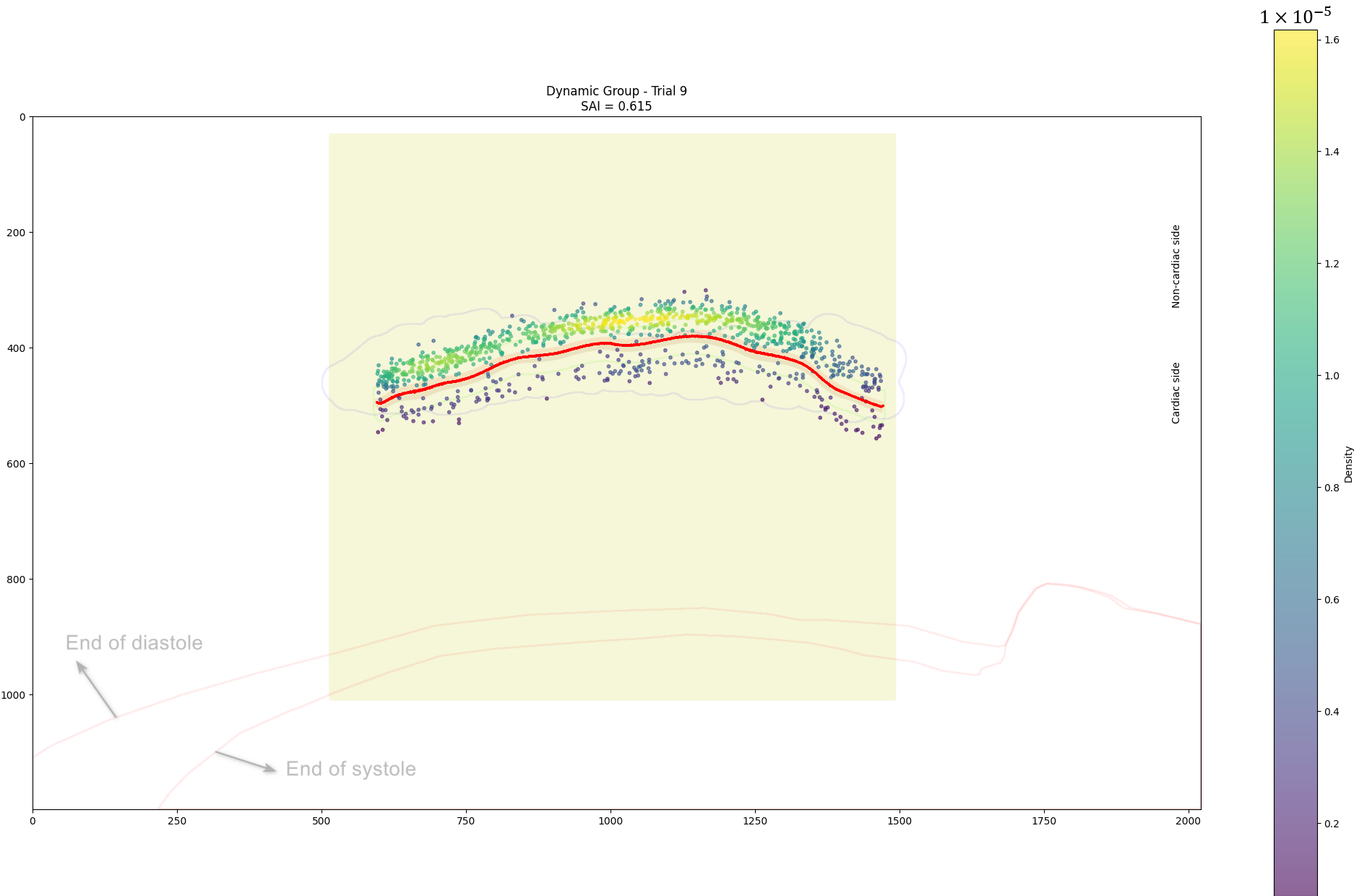

Supplement: Supplementary file 1 [file bioengineering-12-00285-s001.zip › Static and Dynamic groups/dynamic/dynamic_trial_9.png]

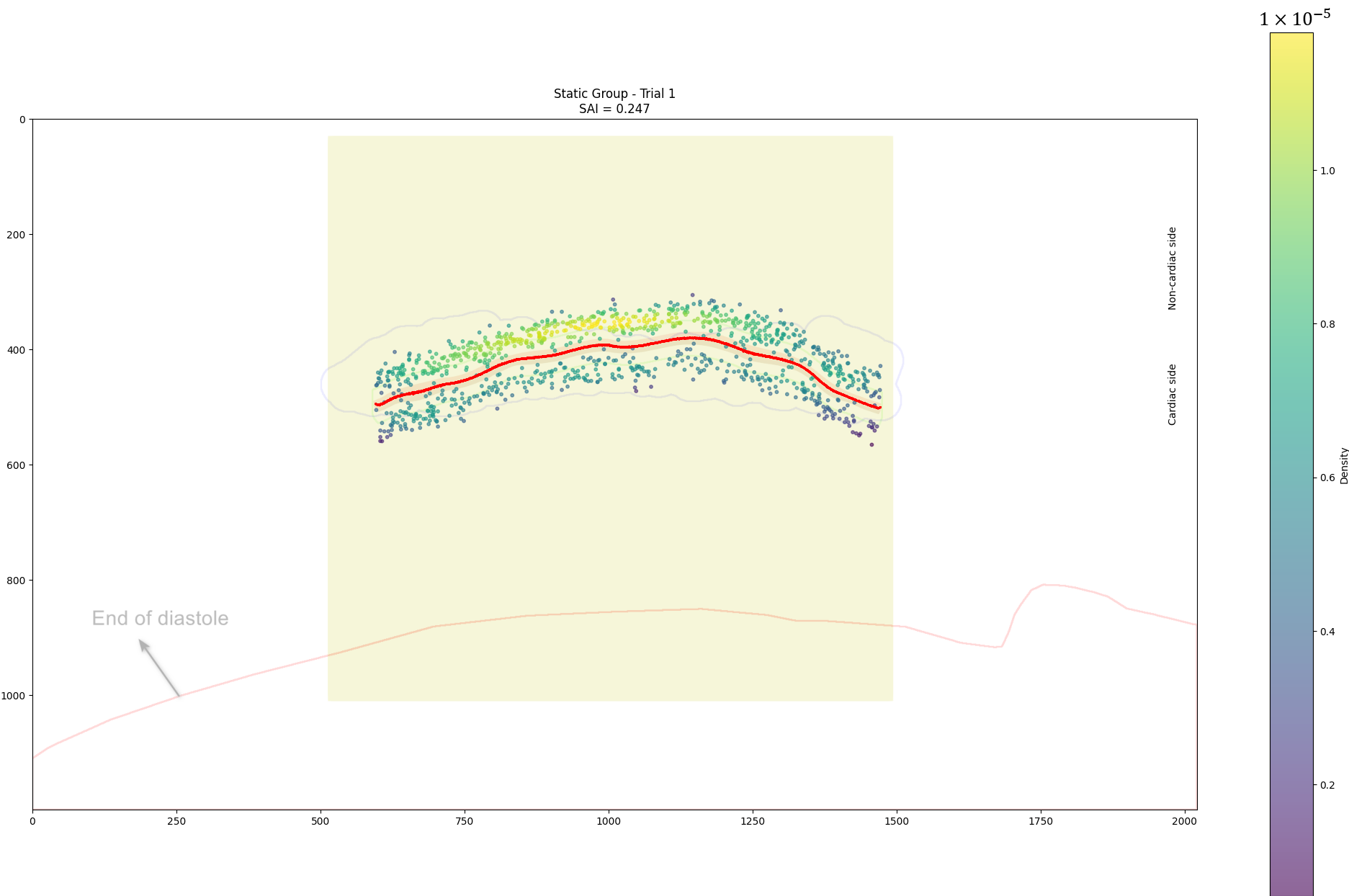

Supplement: Supplementary file 1 [file bioengineering-12-00285-s001.zip › Static and Dynamic groups/static/static_trial_1.png]

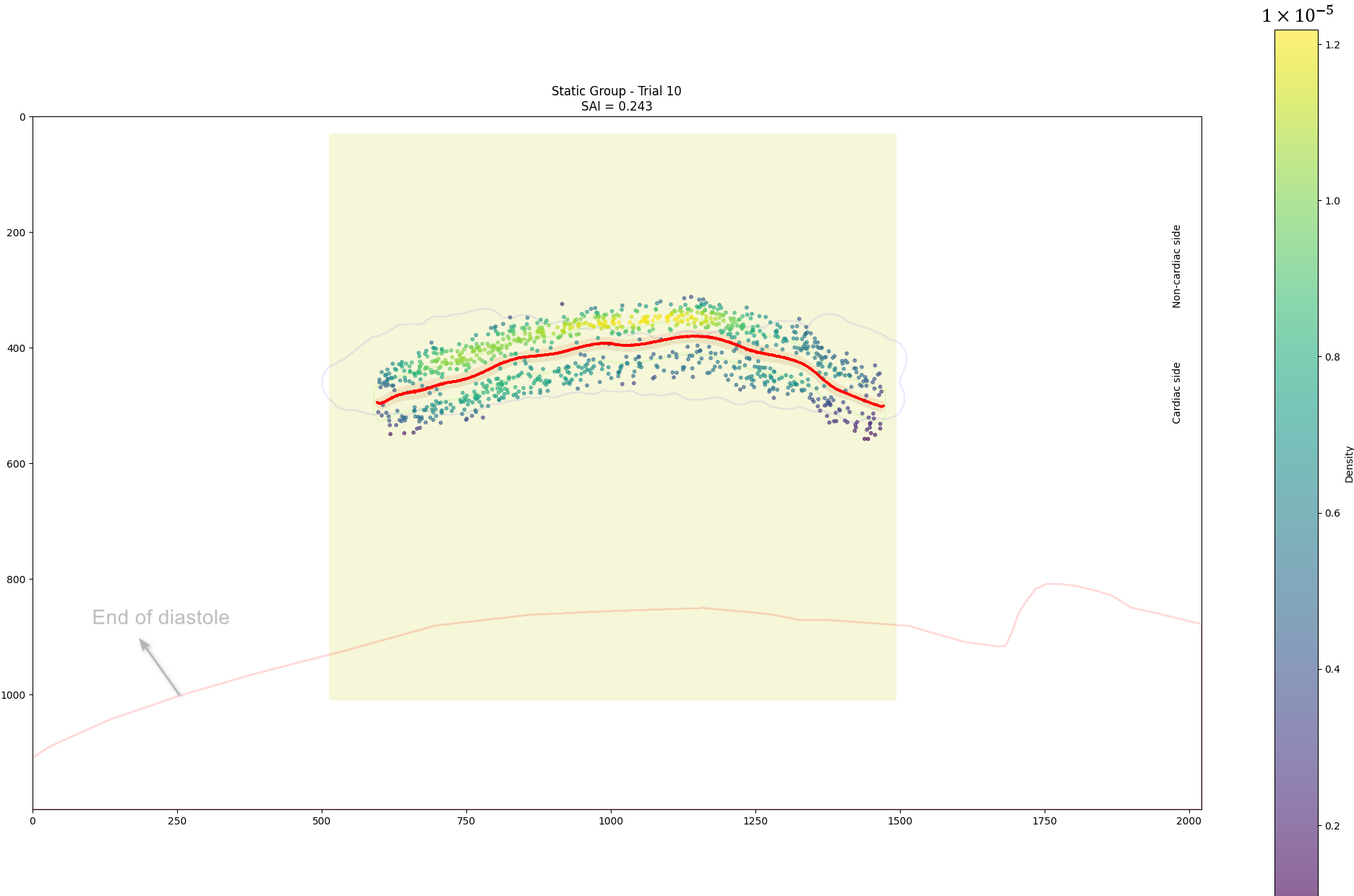

Supplement: Supplementary file 1 [file bioengineering-12-00285-s001.zip › Static and Dynamic groups/static/static_trial_10.png]

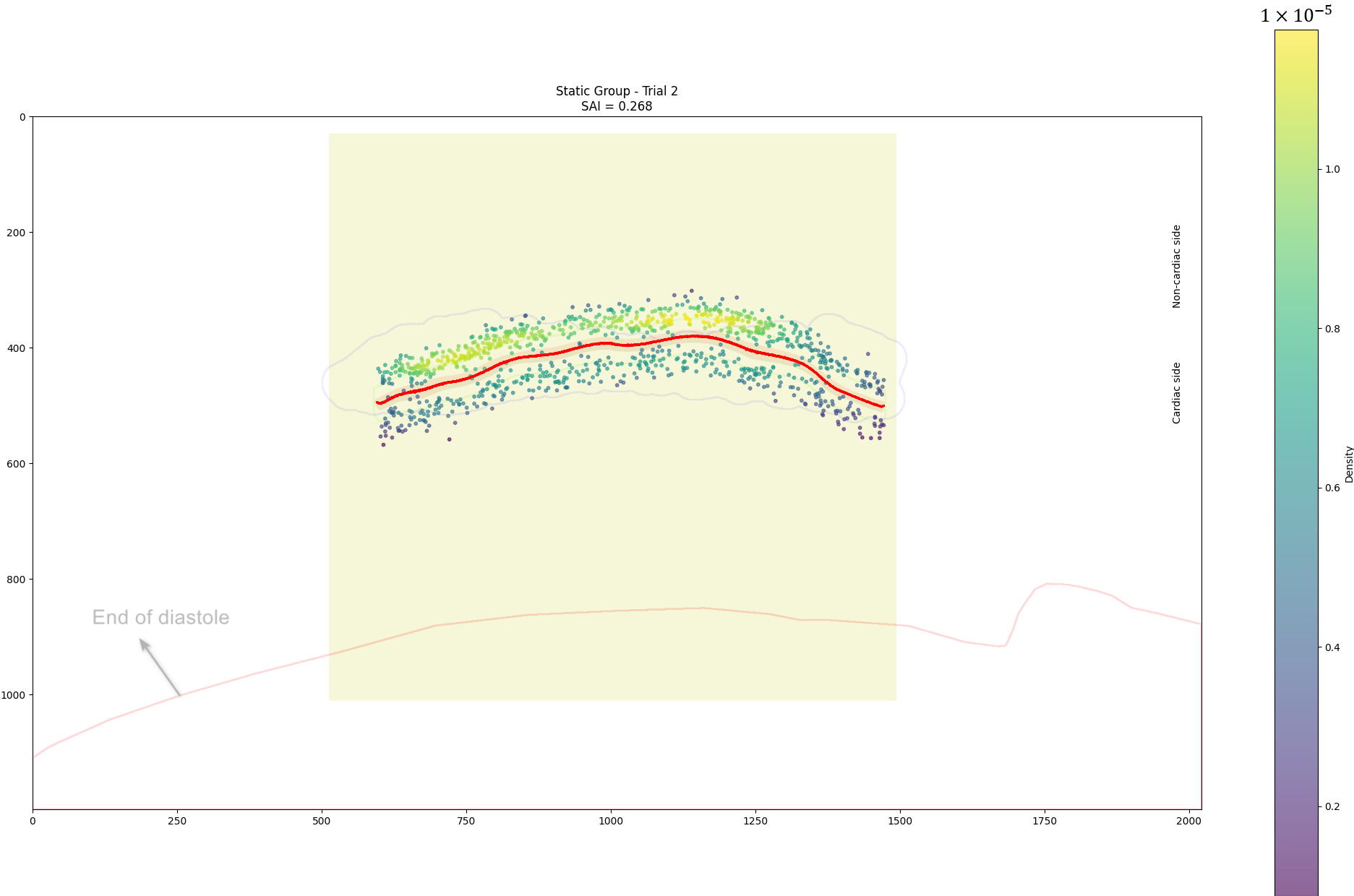

Supplement: Supplementary file 1 [file bioengineering-12-00285-s001.zip › Static and Dynamic groups/static/static_trial_2.png]

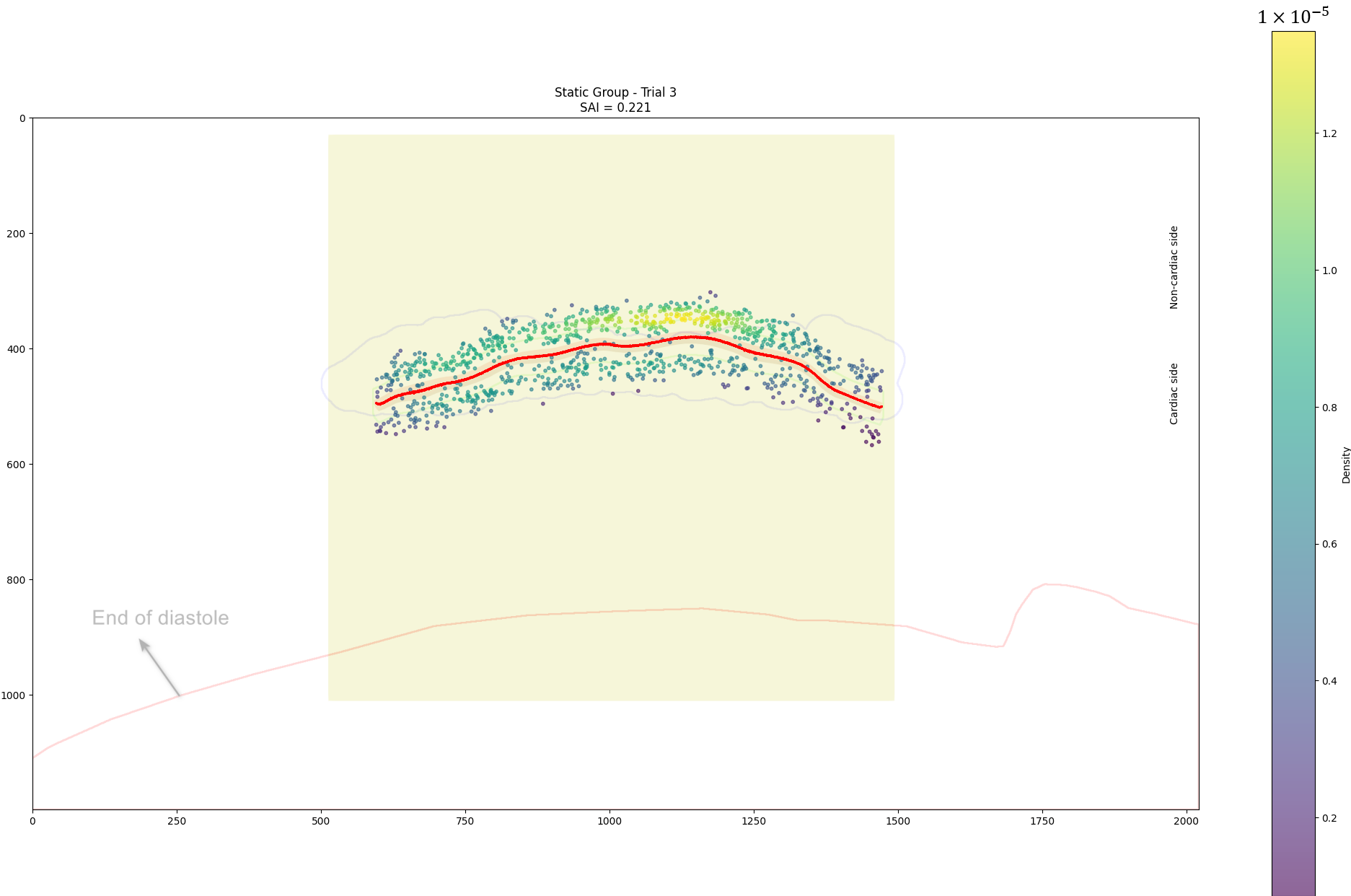

Supplement: Supplementary file 1 [file bioengineering-12-00285-s001.zip › Static and Dynamic groups/static/static_trial_3.png]

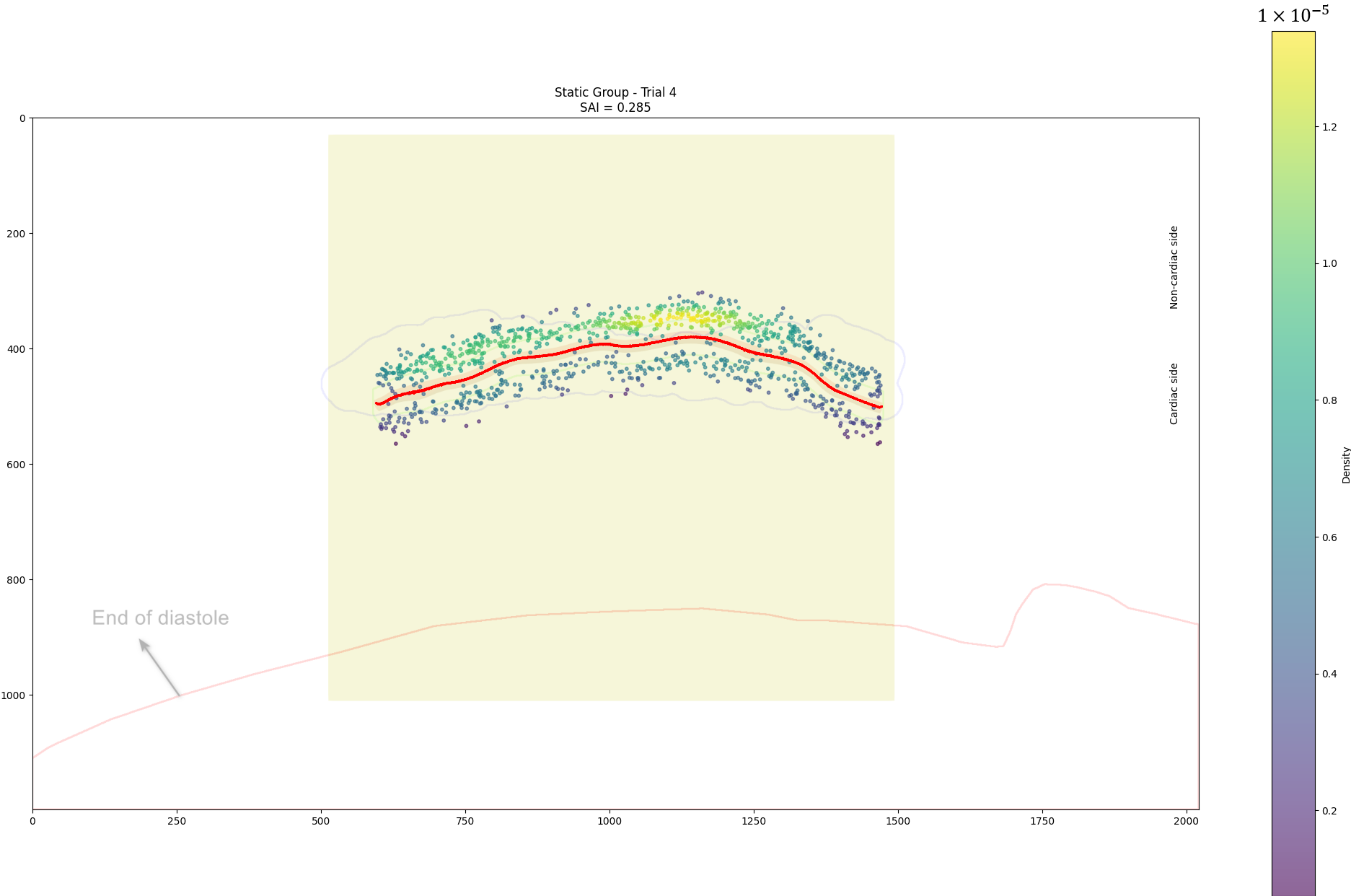

Supplement: Supplementary file 1 [file bioengineering-12-00285-s001.zip › Static and Dynamic groups/static/static_trial_4.png]

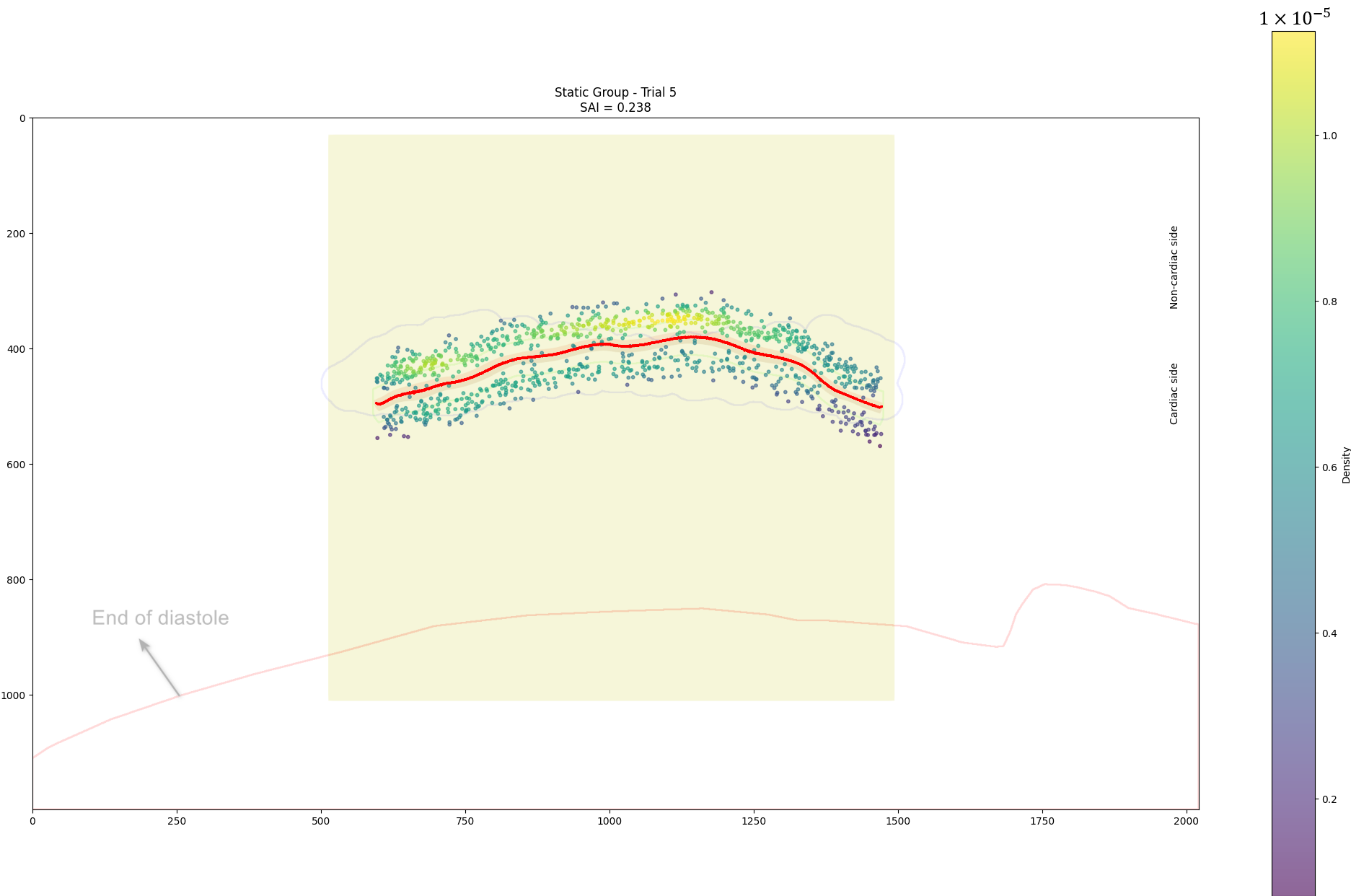

Supplement: Supplementary file 1 [file bioengineering-12-00285-s001.zip › Static and Dynamic groups/static/static_trial_5.png]

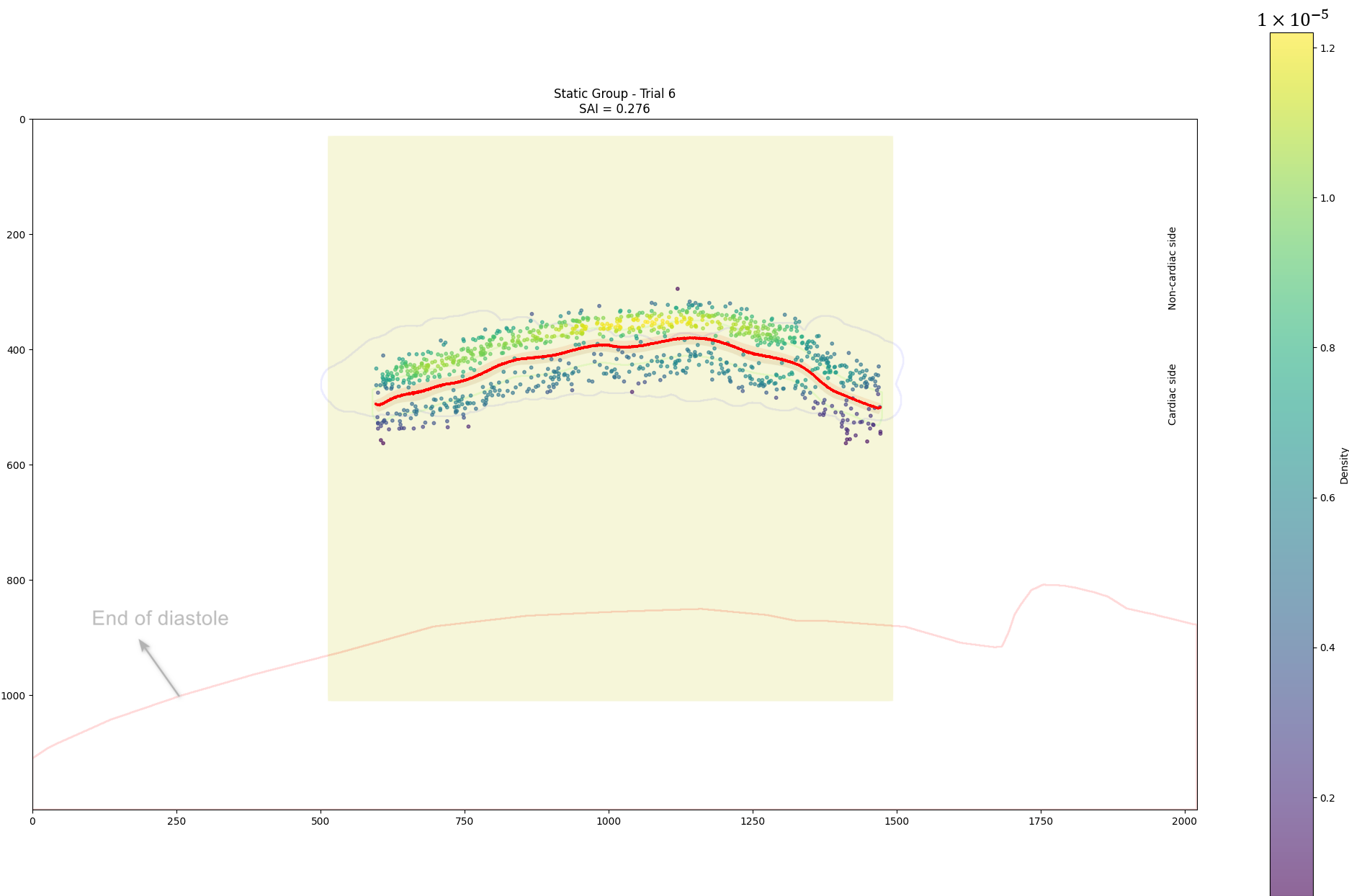

Supplement: Supplementary file 1 [file bioengineering-12-00285-s001.zip › Static and Dynamic groups/static/static_trial_6.png]

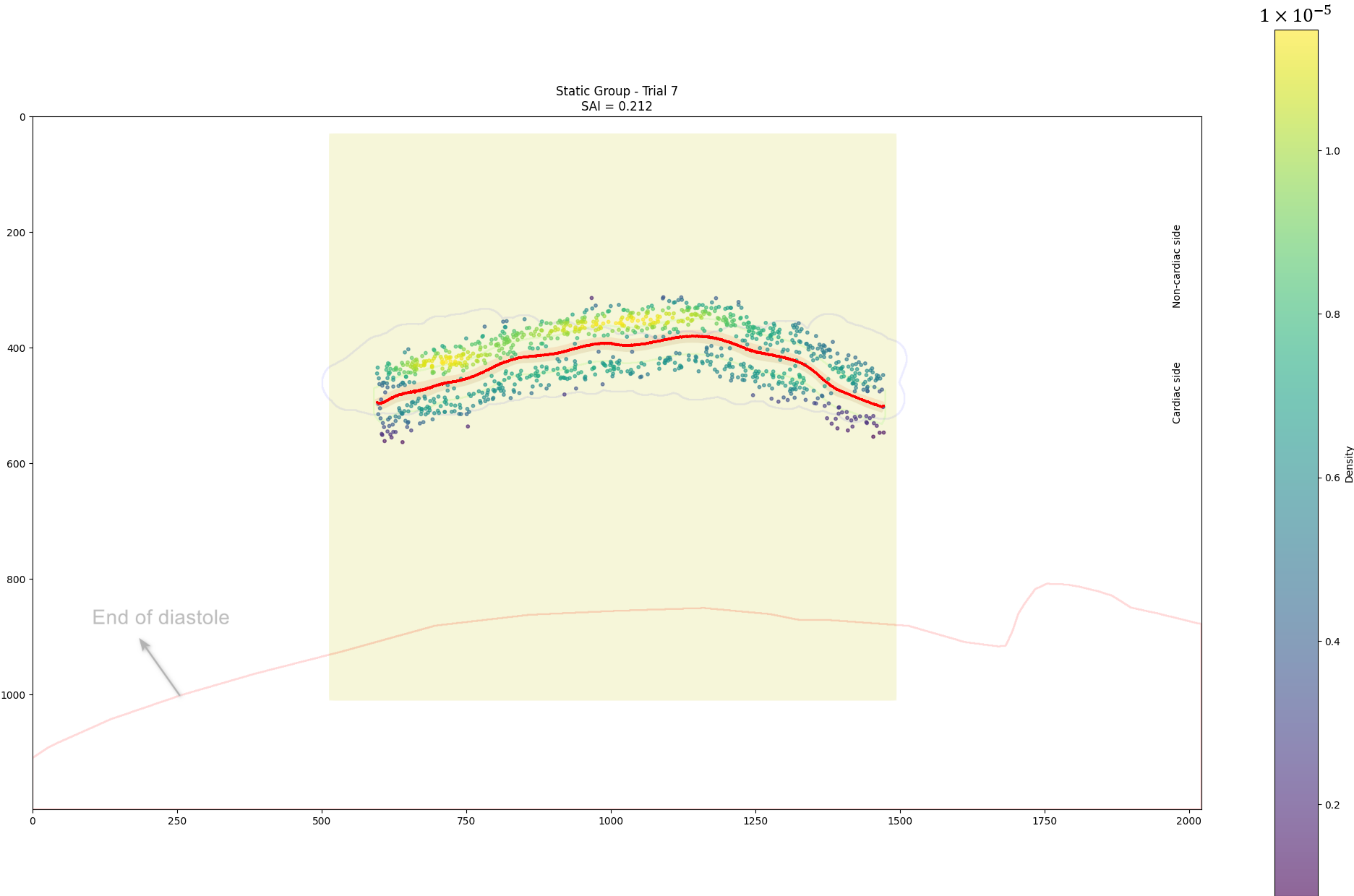

Supplement: Supplementary file 1 [file bioengineering-12-00285-s001.zip › Static and Dynamic groups/static/static_trial_7.png]

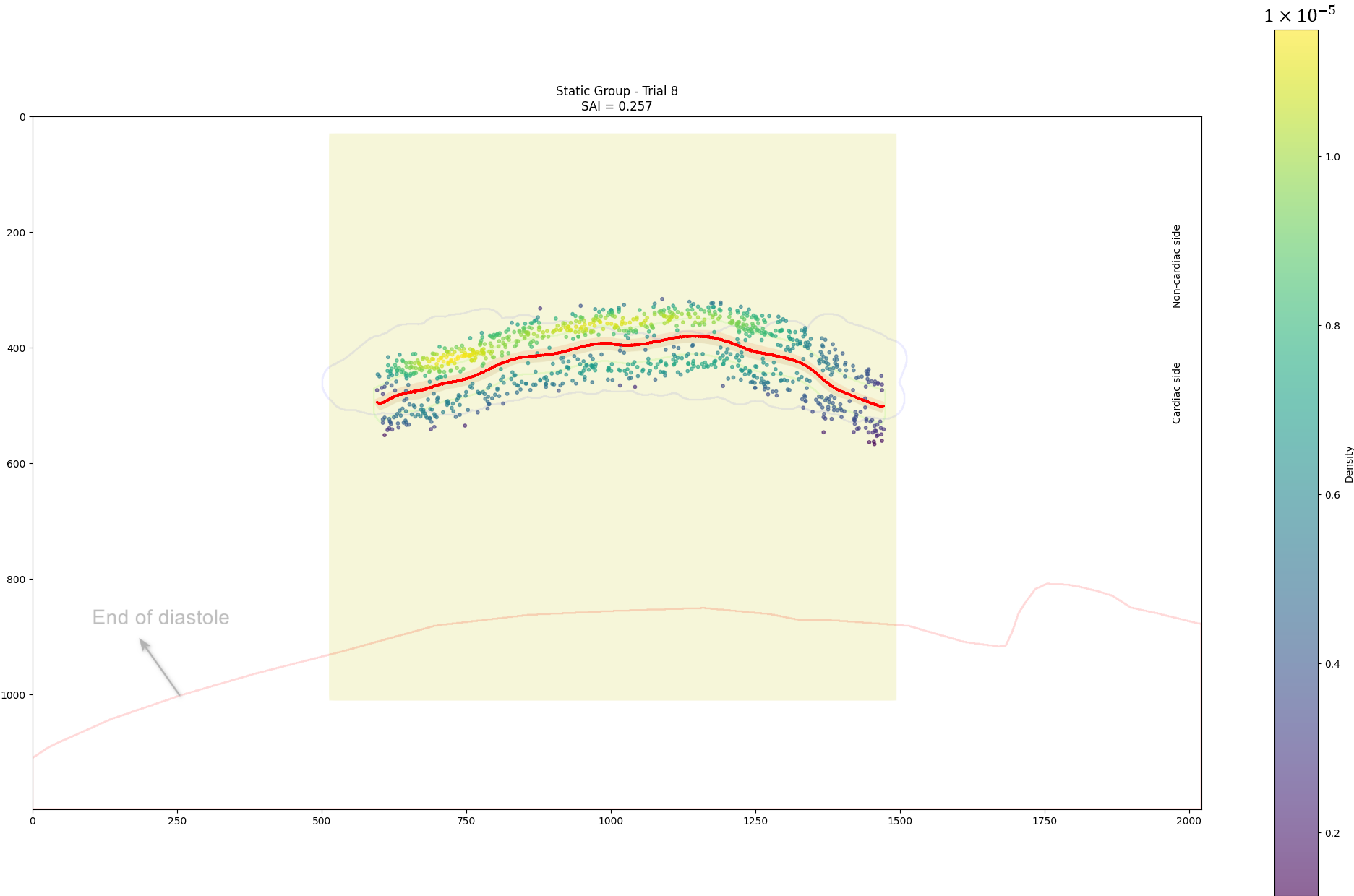

Supplement: Supplementary file 1 [file bioengineering-12-00285-s001.zip › Static and Dynamic groups/static/static_trial_8.png]

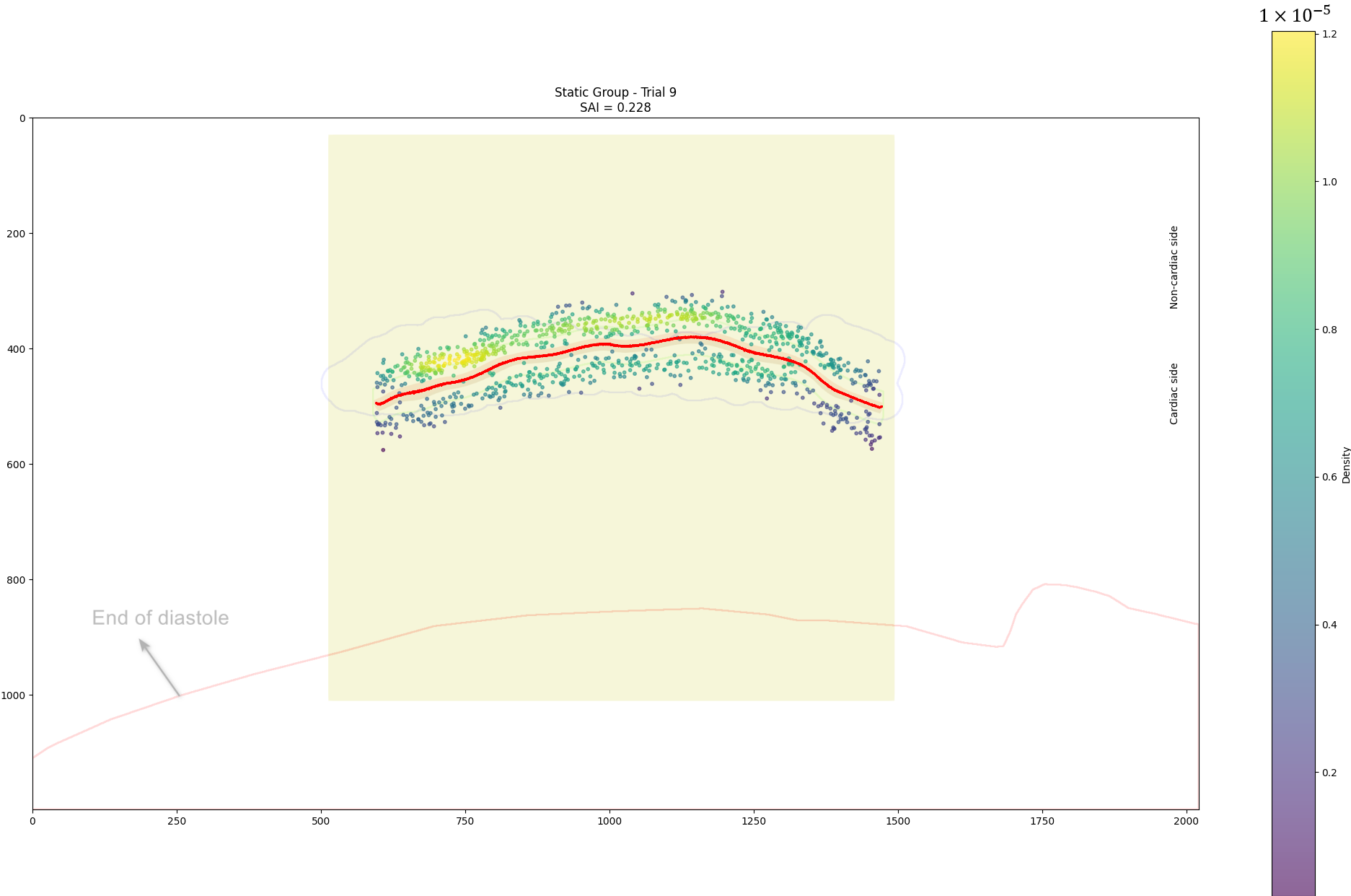

Supplement: Supplementary file 1 [file bioengineering-12-00285-s001.zip › Static and Dynamic groups/static/static_trial_9.png]
